# State of Children Environmental Health Research in Latin America

**DOI:** 10.29024/aogh.908

**Published:** 2018-07-27

**Authors:** Lizbeth López-Carrillo, Leonel González-González, Maricela Piña-Pozas, Ángel Mérida-Ortega, Brenda Gamboa-Loira, Julia Blanco-Muñoz, Luisa E. Torres-Sánchez, Magali Hurtado-Díaz, Marlene Cortez-Lugo, Germán Guerra, Nelly Salgado de Snyder, Mariano E. Cebrian

**Affiliations:** 1 National Institute of Public Health, Cuernacava, Morelos, MX; 2 Department of Toxicology, CINVESTAV, Mexico City, MX

## Abstract

**Background:**

Promotion of biomedical research along with the development of evidence-based prevention policies have been suggested as an effective way to reduce environmental risks for children’s health in Latin America. However, there is little information on the current state of childhood environmental health research, which might help identify its strengths and limitations, as well as to design a strategy to improve the future of child environmental health research in the region.

**Objective:**

To describe the current state of environmental health research on children exposed to environmental pollutants in Latin America.

**Methodology:**

We performed a comprehensive search of published peer-reviewed environmental health articles (1994–2014), dealing with the exposure of Latin American children to chemical compounds. We described the type of studies and their research topics, and identified networks of co-authors. We also analyzed the relationship between research funding sources and the impact factor (IF) of the journal where research was published.

**Results:**

The average number of publications was about 20 per year. Mexico and Brazil produced almost 70% of the 409 identified papers. The most studied contaminant was lead, but research on this element has declined since 2005. Retrospective studies were the most frequent, and also showed a decreasing trend. Most studies did not assess health effects. Four groups of leading investigators and two collaboration models for scientific production were identified. Except for Mexico, there was very little collaboration with North American and European countries. Compared to articles that did not report financial support, those that received international funding had on average an IF around 7, and those with national funding reached a mean IF near 3.

**Conclusion:**

There is a limited number of publications and insufficient collaboration between Latin-American scientists. It is necessary to identify strategies to stimulate South-South-North alliances and strengthen the scarce research on the environmental health of children in the region.

## Introduction

Promotion of biomedical research and development of evidence-based prevention policies, among other strategies, have recently been suggested as a mean to improve children’s environmental health in Latin America [1]. However, there is no current information on the state of childhood environmental health research, which might help identify its strengths and limitations, as well as to consolidate the future of child environmental health research in the region.

Child environmental health problems result from the interaction of several factors, mainly of social and economic origin, which present important contrasts at the global level. The access to safe drinking water, the use of cleaner fuels, and the adoption of personal hygiene and basic sanitation practices largely determine the health of children residing in the poorest countries, whereas reducing chemical exposure is a challenge to preserve and improve children’s health in more developed countries [2].

In most areas of Latin America, basic sanitation environmental risks converge with modern pollution threats, often in a context of poverty and malnutrition. As a result, there is still considerable mortality and morbidity rates in children under five due to infectious diseases and preventable conditions such as pneumonia. In addition, other risks associated with chemical exposure and environmental degradation are also present, such as birth defects, neurodevelopmental disorders, asthma, obesity, diabetes, cardiovascular diseases, mental problems, childhood cancer etc. It is estimated that about 100,000 children under five years of age die each year in America (including USA and Canada) due to physical, chemical and biological environmental hazards [3,4,5,6].

Our purpose is to describe the current state of environmental health research in Latin American children exposed to chemicals by describing some characteristics of published articles on the subject, the definition of the network of co-authors responsible for publication, as well as the analysis of the relationships between research funding sources and their publication in scientific journals of greater international impact.

## Material and methods

We performed a comprehensive literature review through a search of the databases contained in PubMed, SciELO and Lilacs in order to identify and collect articles concerning Latin American children exposed to environmental pollutants and published in Spanish or English during the period from June 1994 to June 2014.

**Figure 1:**
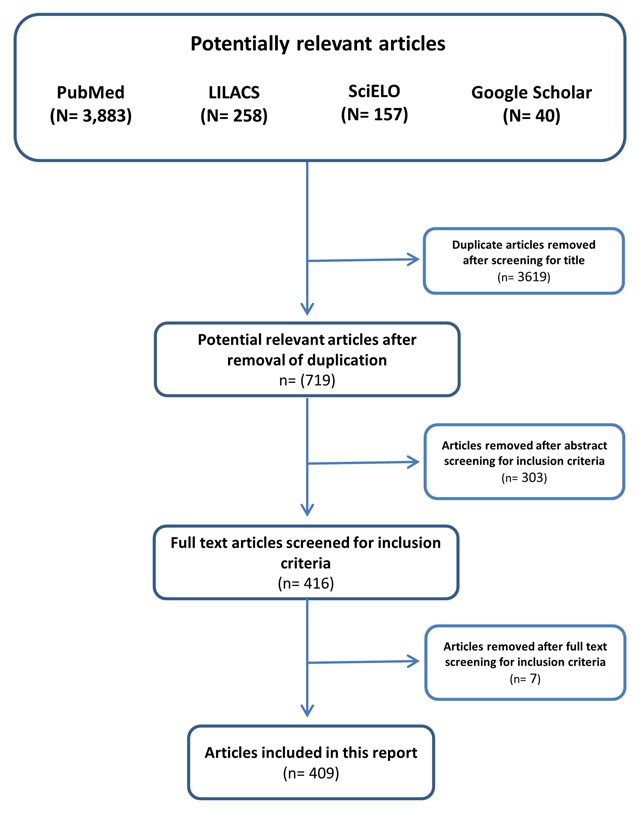
Literature search flow diagram.

Using the same keywords for the three databases (Appendix 1), a total of 4,338 articles were identified (3,883 in Pubmed, 258 in Lilacs, 157 in Scielo, and 40 in Google Scholar). Based on the titles of the articles, 3,619 duplicates were eliminated. Each of the remaining 719 articles was reviewed by two investigators of our team to verify the following inclusion criteria: epidemiological studies carried out in Latin America, with at least 30 subjects younger than 15 years, containing assessment of exposure to chemical contaminants (except tobacco). As a result, 303 items were removed, leaving 416 potentially eligible manuscripts, from which seven additional papers were removed after full text screening for inclusion criteria, yielding a final sample size of 409 manuscripts included in this report (Figure 1).

### Characteristics of the manuscripts

Articles were reviewed to extract the following information: title of the article, name and institution of first and corresponding authors, journal’s name and impact factor (according to Journal Citation Report of 2014), year of publication, country (ies) where the study was carried out, type of funding (none, only national, with international support). We also collected information regarding retrospective or prospective study design (ecological, panel, cross-sectional, case-control, case-cohort, nested case-control, cohort, non-randomized or randomized trials), type of contaminant (ozone, suspended particulates (PM), carbon oxides (CO’x), sulfur oxides (SO’x), nitrogen oxides (NO’x), metals, polycyclic aromatic hydrocarbons (PAHs), volatile organic compounds (VOCs), organochlorines and/or organophosphate pesticides, phthalates, etc.), matrix where exposure was evaluated (water, air, blood, urine, breast milk, hair, saliva, tooth and other human tissues), including assessment by questionnaire, biomarkers or health effects, confounders that were controlled for, and main results. The above information is presented in tables and descriptive charts.

### Network of co-authors

We generated a co-authors square matrix based on the names of the main and corresponding authors by means of the software UCINET 6.314 and its visualization tool NetDraw 2.099 (Analytic Technologies, Harvard, MA) to graphically display the matrix. The latter program allows diagrams of nodes or dots and lines representing main and correspondence authors, as well as lines and arrows showing the directionality of the relationships. As a first step, we applied the “node repulsion and equal edge length bias” method that organizes and distributes the nodes, so that those at a smaller distance are related, while avoiding the crossing and the overlapping of lines between unrelated nodes [7].

We further carried out a faction analysis showing a set of nodes forming a separate structure within a larger network. This method uses a *tabu* search procedure to optimize the minimization of the ratio magnitude until an optimal number of subgroups or factions are found. Then, the “subgroups” and “factions” options were selected in the “analysis” section. As a result, the number of desired central groups appeared, the default option of at least two interrelated nodes and “speed” was chosen as the priority criteria. In the resulting diagram, the factions or subgroups forming independent structures with at least two corresponding authors, and with more than one collaboration between them were marked with a letter, that is, they formed research groups. Finally, we edited the diagram and exported it to a jpeg format word processor [8,9,10].

### Relationships between funding sources and journal’s impact factor

We used multiple linear regression to assess the relationship between the type of funds that supported the research and the impact factor of the journals where articles were published. The distribution of impact factors was normalized by logarithmic transformation. The manuscript characteristics mentioned above were considered as co-variables and remained for further analysis if they resulted statistically significant (p < 0.05). The analysis was performed with the statistical package STATA 14 (StataCorp, College Station, TX, USA). The project was approved by the Ethics Committee of the Mexico National Institute of Public Health.

## Results

In a search of 20 years of research, we found 409 articles dealing with children exposed to chemicals in Latin America – approximately about 20 per year. The highest proportion of articles came from Mexico 46.70 (n = 191), followed by Brazil 21.03 (n = 86), and to a lesser extent from 20 other countries in the region: Ecuador (n = 27), Chile and Nicaragua (n = 7 each), Bolivia (n = 5), Costa Rica (n = 3), Dominican Republic and Trinidad and Tobago (n = 2 each), Belize, Haiti, Honduras and Suriname (n = 1 each) (information not shown in tables).

The most studied pollutant was lead, with about 31% (126/409) of articles, followed by 27.6% on various inorganic compounds (arsenic, fluorine, manganese, iron, selenium, zinc, mercury, chromium, nickel, silver, gold, cadmium, barium, cobalt, and molybdenum) and 25.9% on atmospheric pollutants (ozone, PM, carbon oxides, sulfur oxides, nitrogen oxides, polycyclic aromatic hydrocarbons and volatile organic compounds). Organochlorines and organophosphate pesticides and other organic compounds (polychlorinated and polybrominated compounds, phthalates and petroleum hydrocarbons) were studied in a lower proportion (Table 1).

**Table 1:** Epidemiological articles with Latin American children, according to country and chemical compound 1994–2014 (n = 409).

Chemical compounds	Country	(n)	References (Appendix 2)

Only lead	Mexico	63	(1–63)
	Brazil	19	(64–82)
	Other^1^	44	(83–126)
Inorganic*	Mexico	37	(127–163)
	Brazil	37	(164–200)
	Other^2^	39	(201–239)
Air pollutants**	Mexico	51	(240–290)
	Brazil	25	(291–315)
	Other^3^	30	(316–345)
Organochlorines	Mexico	31	(346–376)
	Brazil	4	(377–380)
	Other^4^	8	(381–388)
Organophosphates	Mexico	5	(389–393)
	Ecuador	5	(394–398)
	Other^5^	3	(399–401)
Organic***	Mexico	4	(402–405)
	Other^6^	4	(406–409)

* Arsenic, fluorine, manganese, iron, selenium, zinc, mercury, chromium, nickel, silver, gold, cadmium, barium, cobalt, lead, molybdenum. ** Ozone, PM, carbon oxides, sulfur oxides, nitrogen oxides, polycyclic aromatic hydrocarbons, volatile organic compounds. *** Polychlorinated and polybrominated compounds, phthalates, petroleum hydrocarbons. ^1^ Ecuador, Chile, Uruguay, Peru, Colombia, Jamaica, Argentina, Venezuela, Belize, Bolivia, Dominican Republic, Trinidad and Tobago. ^2^ Chile, Ecuador, Venezuela, Bolivia, Jamaica, Nicaragua, Peru, Argentina, Colombia, Costa Rica, Suriname, Trinidad and Tobago, Uruguay. ^3^ Guatemala, Chile, Colombia, Ecuador, Peru, Dominican Republic, Haiti, Argentina. ^4^ Nicaragua, Chile, Colombia, Costa Rica, Cuba, Honduras. ^5^ Argentina, Costa Rica, El Salvador. ^6^ Brasil, Chile, Ecuador, Nicaragua.

**Figure 2:**
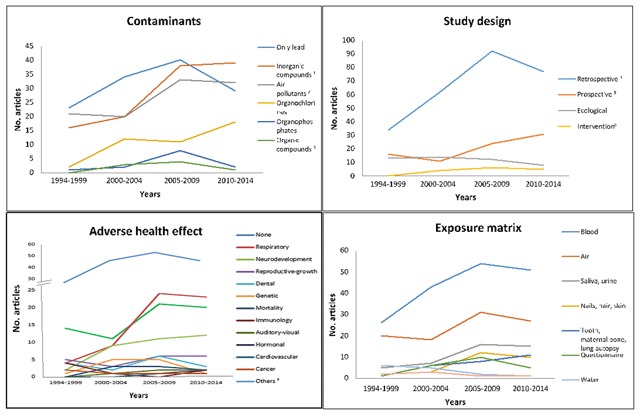
Articles of environmental child’s health by year and selected characteristics (n = 409). ^1^ Arsenic, fluorine, manganese, iron, selenium, zinc, mercury, chromium, nickel, silver, gold, cadmium, barium, cobalt, lead, molybdenum; ^2^ Ozone, PM, carbon oxides, sulfur oxides, nitrogen oxides, polycyclic aromatic hydrocarbons, volatile organic compounds; ^3^ Polybrominated compounds, phthalates, petroleum hydrocarbons; ^4^ Retrospective panel cross-sectional, case-control, retrospective cohort; ^5^ Prospective panel, case-cohort, nested case-control, prospective cohort; ^6^ Randomized and non-randomized trials; ^7^ Erythro porphyrin levels, bilirubin, calcium absorption, cholinesterase activity, biomarkers of inflammation.

Figure 2 shows that despite the high frequency of publications on lead exposure, from 2005 onwards the annual number of publications decreased, similar to studies evaluating exposure to organophosphorus pesticides and volatile organic compounds. This contrasts with the growing trend in the number of studies on organochlorine compounds and the recent plateau of studies on inorganic compounds and air pollutants. Regarding the type of studies, retrospective studies were the most frequent, but starting in 2005, retrospective studies also demonstrated a decreasing trend. In contrast, prospective studies showed an increasing frequency from the beginning of this century. Ecological and intervention studies have been by far the least frequent and have remained almost constant. In many studies (n = 172), children’s health was not assessed, either clinically nor through biomarkers, and these type of studies have also decreased in recent years. Neurological and respiratory harms were the most frequently evaluated health damages and showed a constant trend in the last decade, with blood being the most commonly used matrix to assess exposure.

The impact factor of the journal where articles were published ranged from 0.177 to 45.217 but about half of the articles (50.9%) were published in journals with an impact factor lower than 3.0 (data not included in tables). The type of funding, study design and assessment of health adverse effects were significant predictors of the journal’s impact factor. Compared to articles that did not report financial support, those that received international funding had on average an impact factor around 7 (antilog 2.01), while those with national funding reached a mean impact factor near 3 (antilog 1.21), regardless of whether they were prospective studies or if they had assessed damage to children’s health (Table 2).

**Table 2:** Variables associated with journal impact factor^a^ where articles were published.

Variables	(n)	β	p value

Financial support			
None^b^	(91)	Ref.	
National	(124)	1.21	0.10
International	(194)	2.01	0.00
Study Design			
Retrospective^c^	(312)	Ref.	
Prospective^d^	(97)	1.34	0.00
Health effects			
No	(172)	Ref.	
Yes	(237)	1.33	0.00

^a^ Log-transformed. ^b^ Authors did not report any financial support. ^c^ Ecological, Cross-sectional, retrospective panel, case-control, retrospective cohort. ^d^ Prospective panel, case–cohort, nested case-control, prospective cohort, randomized and non-randomized trials.

**Figure 3:**
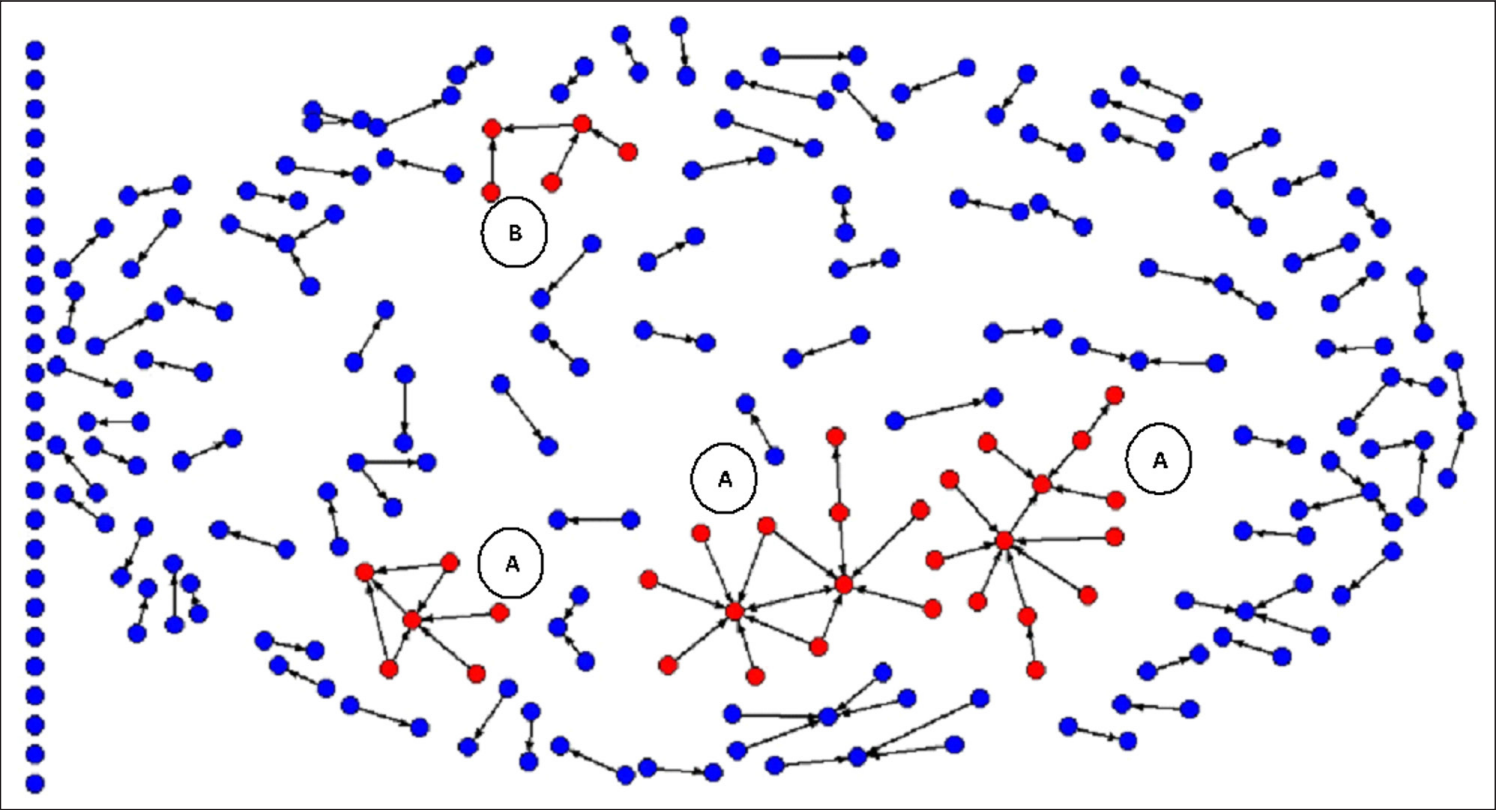
Network of first and corresponding authors in 409 articles on children environmental health in Latin America. Arrows start from the first to the corresponding author. Letters indicae models of collaboration: A) Centralized, B) Distributed.

Figure 3 shows the 367 main and/or corresponding authors. Four co-authors groups (factions) are presented in red. Of those groups, two have three or more corresponding authors and the remaining groups have only two authors. We distinguished two models of collaboration: (A) centralized; characterized by a concentration of the corresponding authorship, and (B) distributed; where main authorship and correspondence is shared alternately between subnet members. In addition, there were 86 subgroups disconnected from each other, consisting of two and up to five nodes. On the left side of Figure 3, there are isolated vertical nodes representing 174 authors who signed as both, first and corresponding authors in a total of 242 articles.

**Figure 4:**
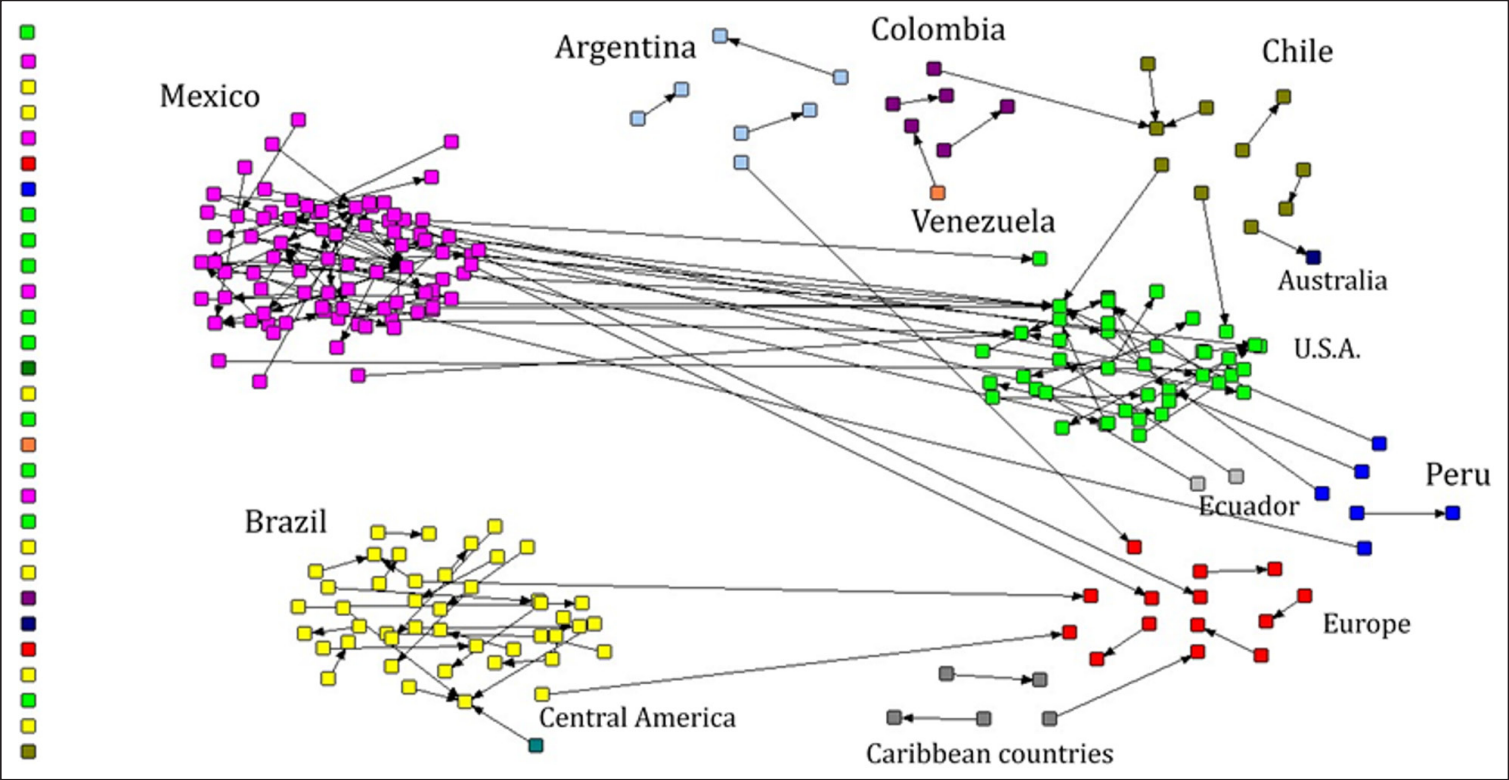
Network of authors and correspondents in 409 articles on children’s environmental health in Latin America, by country. Arrows start from the first to the corresponding author.

Figure 4 shows the collaborative networks in relation to the country of institutional affiliation of first authors. Four models of publication were observed: The first of international character, which prevails in Mexico, is preferentially oriented towards the USA. The second model is co-authoring between same country institutions with very little collaboration abroad, and it was predominant in Brazil and other Latin American countries. In the third model, main and corresponding authorships belong only to authors from the same country. Finally, a fourth model observed that the majority of the 242 articles where the main and corresponding author are the same person were from USA, Mexico and Brazil. It is important to note that the findings reported here refer only to the relationship between first and the corresponding author, but it does not exclude the possibility that in the articles, other researchers whose institution of adscription belongs to a third country participated as secondary co-authors.

## Discussion

Our analysis shows that in the period 1994–2014, Latin America produced a small number of published articles dealing with research on children’s environmental health (about 20 per year), which was mostly implemented in Mexico and Brazil (70% of the total). Studies were predominantly on lead and retrospective in design. The existence of international funding was a significant predictor for publication in major impact journals, regardless of study design.

The most studied pollutant in Latin America is lead. Children are exposed to this metal, due to its use in the production and recycling of batteries, paints, varnishes and poor control of disposal and recycling of e-waste [11]. The articles have evidenced not only the body burden of lead but also its adverse effects, such as delayed neurodevelopment [12,13,14,15,16]. Globally, it has been estimated that childhood lead exposure (>10 µg/dL) annually contributes to at least 600,000 new cases of intellectual disabilities and more than 7,000,000 DALYs due to IQ deficits, corresponding to 80% of the disease burden from lead in the total population, including adults [17]. To date, there is no environmental monitoring system available to detect children at risk from exposure to environmental pollutants in Latin America. The deficiencies of systematic data collection in Latin America are a fundamental barrier to better understand the links between environment, health and sustainable development in the region [18]. Exposure to other inorganic compounds such as arsenic and mercury, as well as indoor and outdoor air pollution, followed in quantity to the number of articles on lead. Most of these articles came from retrospective studies, but did not include the evaluation of adverse health effects and were limited in their scope.

The great research heterogeneity in Latin American countries is evidenced by the relatively low number of articles published on children environmental health, which is consistent with the condition of other research topics [19]. Although the investment in science in each country is a strong determinant for scientific production, the national approach to collaboration should also be considered. In this regard, in the last two decades funding for scientific research has increased in many, but not all, Latin American countries. For example, the economic power of Brazil tripled and its scientific production in general increased five-fold. However, considering the number of articles per capita, Brazil has a production similar to Argentina, Uruguay and Chile [19]. On the subject of children’s environmental health, our results showed that Brazil has a research model concentrated in itself, with marginal collaboration with Europe and other Latin and North American countries. This situation is consistent with Brazil’s deficit of international collaboration in other areas of science, such as clinical medicine, where it shows little collaboration with South American countries [20].

In addition, it is important to consider that a large number of articles on children’s environmental health in the region is produced by few research groups with mostly centralized collaboration networks. Although these groups have a strong leadership, are highly organized, and have high production, they tend to limit the freedom for action of their members, perhaps compromising the advancement of investigators in the network periphery, contrary to what happens in other research models where participation and joint development of all members is stimulated [21].

Some authors have suggested making children a priority research target in transdisciplinary approaches, including basic, clinical and public health research would help to find solutions to specific problems [22]. In particular, we consider it important to develop transdisciplinary studies dealing with early childhood exposures in undernourished children. It is estimated that at least 5% of children under five in Latin America suffer from moderate to severe malnutrition [4].

It is also necessary to advance and standardize methods for environmental exposure assessment, with better biomarkers and study designs allowing researchers to estimate with greater certainty the burden of disease associated with exposures not considered in the current Global Burden of Disease project [23]. This may be achieved by creating South-South-North virtual collaboration centers to enhance scientific and technological exchange, as well as promoting less centralized leadership models in order to allow research group members to expand collaboration networks.

Even without definitive evidence of causality, we consider that interventions should be strengthened to reduce environmental exposures associated with adverse health effects. This point is especially important for Latin America, where preventive actions which could not necessarily emanate from scientific evidence developed in the region could be implemented. At this point, it is important to emphasize that children are a population especially vulnerable to the adverse effects of exposure to pollutants. Children’s recreational activities expose them to soil and other contact surfaces that are vehicles of harmful substances and the relative magnitude of exposure corresponds to a higher dose if the smaller weight and size of the infants is considered. The same happens with the ingestion of contaminated water and food, their volume and frequency of consumption in relation to the size of a child is greater than in the adult. Moreover, children may have lesser ability to metabolize, detoxify, and excrete toxic chemicals when compared to adults [5].

In conclusion, there is a limited number of publications and insufficient collaboration between Latin American scientists. It is necessary to identify strategies to stimulate South-South-North alliances and to strengthen the scarce research on children’s environmental health in the region. As it has been suggested [24], collaborative research will be the engine that drives environmental health forward in the 21 st century. In this context, it is evident the need to enhance children environmental health research in Latin America, to guide pediatric practice, stimulate advocacy and influence public policy.

## Additional Files

The additional files for this article can be found as follows:

10.29024/aogh.908.s1 Appendix 1. Key words used in the literature review.

10.29024/aogh.908.s2 Appendix 2. References form Table 1.
