# Supplementary material for: State of Children Environmental Health Research in Latin America
Source: Ann Glob Health. 2018 Jul 27;84(2):204–11. doi: 10.29024/aogh.908 (PMC6748241; doi:10.29024/aogh.908)
Supplement: Appendix 1. — Key words used in the literature review. [file agh-84-2-908-s1.pdf]

Appendix 1

Key words used in the literature review

- Children
- Child
- Infant
- Fetus
- Air pollutants
- Soil pollutants
- Water pollutants
- Environmental pollutants
- Aerosol Propellants
- Air Pollution
- Water Pollution
- Tobacco Smoke
- Environmental Pollution
- Agrochemicals
- Aflatoxins
- Alpha-hexachlorocyclohexane
- Alpha-HCH
- Aldrin
- Aluminum
- Ammonia
- Arsenic
- Asbestos
- Benzene
- Biofuels
- Bisphenol A
- BPA
- Cadmium
- Carbamates
- Propoxur
- Carbon Dioxide
- Carbon Tetrachloride
- Carbon Monoxide
- Nitrosamines
- Carcinogens
- Carcinogen
- Charcoal nada lilacs
- Chlordan nada lilacs
- Chlordecone nada lilacs
- Chromium
- Copper
- Cyanides
- Cyanide
- Dieldrin
- Vehicle Emissions
- Vehicle Emission
- Gasoline
- Dichlorodiphenyl
- Dichloroethylene
- DDT
- Dioxins
- Dioxin
- Waste Water
- Drinking Water
- Dust
- Coloring Agents
- Coloring Agent
- Electronic Waste
- Electronic Wastes
- Endrin
- Endocrine Disruptors
- Endocrine Disruptor
- Endosulfans
- Endosulfan
- Environmental Exposures
- Environmental Exposure
- Fertilizers OR Fertilizer
- Fluorides OR Fluoride
- Formaldehyde
- Fungicides
- Industrial
- Fungicides
- Fungicide
- Furans
- Hazardous Substances
- Hazardous Substance
- Metals OR Metal
- Heptachlor nada
- Herbicides
- 2,2',4,4',5,5'-hexabrominated diphenyl
- Hexabromocyclododecane
- HBCD
- Hexachlorobenzene
- Hydrocarbons
- Hydrocarbon
- Insecticides
- Lead
- Lindane
- Malondialdehyde
- Medical Waste
- Mercury
- Methylmercury Compounds
- Mirex
- Natural Gas
- Nicotine
- Cotinine
- Nickel
- Nitrates
- Nitrous Oxide
- Occupational Exposure
- Organotin Compounds
- Hydrocarbons
- Chlorinated
- Diazinon
- Organothiophosphorus Compounds
- Ozone
- Perfluorooctanoic acid
- PFOA
- Alkenes
- Eethylene
- Propylene
- Butadiene
- Styrene
- Petroleum
- Perfluorooctane sulfonic acid
- PFSO
- Phthalates
- Phthalic acid
- Plasticizers
- Polyvinyl chloride
- PVC
- Polybrominated biphenyls
- Pentabromodiphenyl ether
- Polychlorinated biphenyls
- PCBs
- Chlorinated dibenzofurans
- Polycyclic Aromatic Hydrocarbons
- Pyrethrins
- Radioactive pollutants
- Radon
- Rodenticides
- Selenium
- Sulfur dioxide
- Sulfur oxides
- Soil
- Soil pollutants
- Particulate matter
- Thallium
- Thalidomide
- Tobacco smoke pollution
- Toluene
- Toxaphene
- Volatile organic compounds
- Water pollutants
- Zinc
- 1-hydroxypyrene
- Methylphenidate
- Latin America
- Caribbean countries
- Central America
- Costa Rica
- El Salvador
- Guatemala
- Honduras
- Nicaragua
- Panama
- English-Speaking Caribbean
- Anguilla
- Antigua and Barbuda
- Aruba
- Bahamas
- Barbados
- Belize
- Bermuda
- British Virgin Islands
- UK
- Cayman Islands
- Dominica
- Grenada
- Guyana
- Jamaica
- Netherland Antilles
- Saint Kitts & Nevis
- Saint Lucia
- Saint Vincent & the Grenadines
- Suriname
- Trinidad & Tobago
- Turks and Caicos
- French-Speaking Caribbean
- French Guiana
- Guadeloupe
- Haiti
- Martinique
- Montserrat
- South America
- Argentina
- Bolivarian Republic of Venezuela
- Bolivia
- Brazil
- Chile
- Colombia
- Ecuador
- Paraguay
- Peru
- Uruguay
- Spanish-Speaking Caribbean
- Cuba
- Dominican Republic
- México
