# Supplementary material for: State of Children Environmental Health Research in Latin America
Source: Ann Glob Health. 2018 Jul 27;84(2):204–11. doi: 10.29024/aogh.908 (PMC6748241; doi:10.29024/aogh.908)
Supplement: Appendix 2. — References form Table 1. [file agh-84-2-908-s2.pdf]

Appendix 2

References form Table 1

1. Kordas K, Casavantes KM, Mendoza C, et al. The association between lead and micronutrient status, and children's sleep, classroom behavior, and activity. Arch Environ Occup Health. 2007;62(November 2014):105-112. doi:10.3200/AEOH.62.2.105-112.

2. Manzanares-Acuña E, Vega-Carrillo H, Salas-Luévano M, Hernández-Dávila V, Letechipía-de León C, Bañuelos-Valenzuela R. Niveles de plomo en la población de alto riesgo y su entorno en San Ignacio, Fresnillo, Zacatecas, México. Salud Publica Mex. 2006;48(3):212-219.

3. Olaiz G, Fortoul T, Rojas R, Doyer M, Palazuelos E, Tapia C. Risk factors for high levels of lead in blood of schoolchildren in Mexico City. Arch Environ Health. 1996;51(2):122-126.

4. Gomaa A, Hu H, Bellinger D, et al. Maternal Bone Lead as an Independent Risk Factor for Fetal Neurotoxicity: A Prospective Study. Pediatrics. 2002;110(1):110-118. doi:10.1542/peds.110.1.110.

5. Hopkins MR, Ettinger AS, Hernández-Avila M, et al. Variants in Iron Metabolism Genes Predict Higher Blood Lead Levels in Young Children. Environ Health Perspect. 2008;116(9):1261-1266. doi:10.1289/ehp.11233.

6. Kordas K, Stoltzfus RJ, López P, Alatorre Rico J, Rosado JL. Iron and zinc supplementation does not improve parent or teacher ratings of behaviour in first grade Mexican children exposed to lead. J Pediatr. 2005;147:632-639. doi:10.1016/j.jpeds.2005.06.037.

7. Henn BC, Schnaas L, Ettinger AS, et al. Association of early childhood manganese and lead co-exposure with neurodevelopment. Environ Health Perspect. 2012;120(1):126-131. doi:10.1289/ehp.1003300).

8. Jiménez-Gutiérrez C, Romieu I, Ramírez-Sánchez AL, et al. Exposición a plomo en niños de 6 a 12 años de edad. Salud Publica Mex. 1999;41(supl 2):S72-S81.

9. Hernandez-Avila M, Sanin L, Romieu I, et al. Higher milk intake during pregnancy is associated with lower maternal and umbilical cord lead levels in postpartum women. Environ Res. 1997;74:116-121.

10. Ettinger AS, Téllez-Rojo MM, Amarasiriwardena C, et al. Influence of maternal bone lead burden and calcium intake on levels of lead in breast milk over the course of lactation. Am J Epidemiol. 2006;163(1):48-56. doi:10.1093/aje/kwj010.

11. Pilsner JR, Hu H, Wright RO, et al. Maternal MTHFR genotype and haplotype predict deficits in early cognitive development in a lead-exposed birth cohort in Mexico City. Am J Clin Nutr. 2010;92(1):226-234. doi:10.3945/ajcn.2009.28839.

12. Sanin LH, Gonzalez-Cossio T, Romieu I, et al. Effect of Maternal Lead Burden on Infant Weight and Weight Gain at One Month of Age Among Breastfed Infants. Pediatrics. 2001;107(5):1016-1023. doi:10.1542/peds.107.5.1016.

13. Rothenberg SJ, Cansino S, Sepkoski C, et al. Prenatal and perinatal lead exposures alter acoustic cry parameters of neonate. Neurotoxicol Teratol. 1995;17(2):151-160. doi:10.1016/0892-0362(94)00066-M.

14. Schnaas L, Rothenberg SJ, Perroni E, Martínez S, Hernández C, Hernández RM. Temporal pattern in the effect of postnatal blood lead level on intellectual development of young children. Neurotoxicol Teratol. 2000;22(6):805-810. doi:10.1016/S0892-0362(00)00101-X.

15. Alatorre-Rico J, Kordas K, López P, et al. Efficacy of Iron and/or Zinc Supplementation on Cognitive Performance of Lead-Exposed Mexican Schoolchildren: A Randomized, Placebo-Controlled Trial. Pediatrics. 2006;117(3):e518-e527. doi:10.1542/peds.2005-1172.

16. Rubio-Andrade M, Valdés-Pérezgasga F, Alonso J, Rosado JL, Cebrián ME, García-Vargas GG. Follow-up study on lead exposure in children living in a smelter community in northern Mexico. Environ Heal. 2011;10(1):66-72. doi:10.1186/1476-069X-10-66.

17. Rothenberg SJ, Schnaas L, Perroni E, Hernandez RM, Karchmer S. Secular trend in blood lead levels in a cohort of Mexico City children. Arch Environ Health. 1998;53(3):231-235. doi:10.1080/00039899809605700.

18. Calderón-Salinas J, Hernandez-Luna C, Valdez-Anaya B, Maldonado-Vega M, Lopez-Miranda A. Evolution of lead toxicity in a population of children. Hum Exp Toxicol. 1996;15(5):376-382. doi:10.1177/096032719601500502.

19. Rothenberg SJ, Schnaas L, Perroni E, Hernández RM, Ortega JF, Flores Ortega J. Blood lead secular trend in a cohort of children in Mexico City. II. 1990–1995. Arch Environ Heal An Int J. 2000;55(4):245-249. doi:10.1080/00039890009603414.

20. Hernández-Guerrero J, Jiménez-Farfán M, Belmont R, Ledesma-Montes C, Baez A. Lead levels in primary teeth of children living in Mexico City. Int J Pediatr Dent. 2004;14(3):175-181. doi:10.1111/j.1365-263X.2004.00536.x.

21. Schnaas L, Rothenberg SJ, Flores MF, et al. Reduced intellectual development in children with prenatal lead exposure. Environ Health Perspect. 2006;114(5):791-797. doi:10.1289/ehp.8552.

22. Hu H, Téllez-Rojo MM, Bellinger D, et al. Fetal lead exposure at each stage of pregnancy as a predictor of infant mental development. Environ Health Perspect. 2006;114(11):1730-1735. doi:10.1289/ehp.9067.

23. Azcona-Cruz MI, Romero-Placeres M, Rothenberg SJ, et al. Lead-Glazed Ceramic Ware and Blood Lead Levels of Children in the City of Oaxaca, Mexico. Arch Environ Health. 2000;55(3):217-222. doi:10.1080/00039890009603409.

24. Kordas K, Canfield RL, López P, et al. Deficits in cognitive function and achievement in Mexican first-graders with low blood lead concentrations. Environ Res. 2006;100(3):371-386. doi:10.1016/j.envres.2005.07.007.

25. Cowan L, Esteban E, McElroy-Hart R, et al. Binational study of pediatric blood lead levels along the United States/Mexico border. Int J Hyg Environ Health. 2006;209(3):235-240. doi:10.1016/j.ijheh.2005.12.003.

26. Rothenberg SJ, Schnaas L, Salgado-Valladares M, et al. Increased ERG a- and b-wave amplitudes in 7- to 10-year-old children resulting from prenatal lead exposure. Investig Ophthalmol Vis Sci. 2002;43(6):2036-2044.

27. López-Carrillo L, Torres-Sánchez L, Garrido F, Papaqui-Hernández J, Palazuelos-Rendón E, López-Cervantes M. Prevalence and determinants of lead intoxication in Mexican children of low socioeconomic status. Environ Health Perspect. 1996;104(11):1208-1211. doi:10.1289/ehp.961041208.

28. Ordóñez B, Ruiz Romero L, Mora R. Investigación epidemiológica sobre niveles de plomo en la población infantil y en el medio ambiente domiciliario de Ciudad Juárez, Chihuahua, en relación con una fundición de El Paso, Texas. Salud Publica Mex. 2003;45(supl 2):S281-S295. http://www.scielo.org.mx/scielo.php?script=sci_arttext&pid=S0036-36342003000800015.

29. Braun JMJ, Hoffman E, Schwartz J, et al. Assessing windows of susceptibility to lead-induced cognitive deficits in Mexican children. Neurotoxicology. 2012;33(5):1040-1047. doi:10.1002/ana.22528.Toll-like.

30. Soto-Jiménez MF, Flegal AR. Childhood lead poisoning from the smelter in Torreón, México. Environ Res. 2011;111:590-596. doi:10.1016/j.envres.2011.01.020.

31. Recio-Vega R, Valdez-Abrego C, Adame-Lopez B, Gurrola-Mendez A. Surveillance of elevated blood lead levels in children in Torreon, Coahuila, Mexico, 1998-2010. Int J Hyg Environ Health. 2012;215(5):507-513. doi:10.1016/j.ijheh.2011.10.009.

32. Kordas K, Ettinger AS, Lamadrid-Figueroa H, et al. Methylenetetrahydrofolate reductase (MTHFR) C677T, A1298C and G1793A genotypes, and the relationship between maternal folate intake, tibia lead and infant size at birth. Br J Nutr. 2009;102:907-914. doi:10.1017/S0007114509318280.

33. Albalak R, McElroy R, Noonan G, et al. Blood lead levels and risk factors for lead poisoning amog children in a Mexican smelting community. Arch Environ Health. 2003;58(3):172-183.

34. Díaz-Barriga F, Batres L, Calderón J, et al. The El Paso smelter 20 years later: residual impact on Mexican children. Environ Res. 1997;74(1):11-16. doi:10.1006/enrs.1997.3741.

35. Kordas K, Lopez P, Rosado JL, et al. Blood Lead, anemia, and short stature are independently associated with cognitive performance in Mexican school children. J Nutr. 2004;134:363-371.

36. Jarrell JF, Weisskopf MG, Weuve J, Téllez-Rojo MM, Hu H, Hernández-Avila M. Maternal lead exposure and the secondary sex ratio. Hum Reprod. 2006;21(7):1901-1906. doi:10.1093/humrep/del047.

37. Navarrete-Espinosa J, Sanín-Aguirre LH, Escandón-Romero C, Benitez-Martínez G, Olaiz-Fernández G, Hernández-Avila M. Niveles de plomo sanguíneo en madres y recién nacidos

derechohabientes del Instituto Mexicano del Seguro Social. Salud Publica Mex. 2000;42(5):391-396.

38. Cantonwine D, Hu H, Téllez-Rojo MM, et al. HFE gene variants modify the association between maternal lead burden and infant birthweight: a prospective birth cohort study in Mexico City, Mexico. Environ Heal. 2010;9:43. doi:10.1186/1476-069X-9-43.

39. Torres-Sánchez L, Berkowitz G, López-Carrillo L, Torres-Arreola L, Ríos C, López-Cervantes M. Intrauterine lead exposure and preterm birth. Environ Res. 1999;81(4):297-301. doi:10.1006/enrs.1999.3984.

40. García Vargas GG, Rubio Andrade M, Del Razo LM, Borja Aburto V, Vera Aguilar E, Cebrián ME. Lead exposure in children living in a smelter community in Region Lagunera, Mexico. J Toxicol Environ Heal - Part A. 2001;62(6):417-429. doi:10.1080/00984100150501150.

41. Schnaas L, Rothenberg SJ, Flores MF, et al. Blood lead secular trend in a cohort of children in Mexico City (1987-2002). Environ Health Perspect. 2004;112(10):1110-1115. doi:10.1289/ehp.6636.

42. Flores-Ramírez R, Rico-Escobar E, Núñez-Monreal JE, et al. Exposición infantil al plomo en sitios contaminados. Salud Publica Mex. 2012;54(4):383-392. doi:10.1590/S0036-36342012000400008.

43. Poblano A, Rothenberg SJ, Schnaas L, Elías Y, Cruz ML. Spatial distribution of EEG theta activity as a function of lifetime lead exposure in 9-year-old children. Neurotoxicology. 2001;22(4):439-446. doi:10.1016/S0161-813X(01)00038-9.

44. Afeiche M, Peterson K, Sánchez B, et al. Windows of lead exposure sensitivity, attained height, and BMI at 48 months. J Pediatr. 2012;160(6):1044-1049. doi:10.1002/ana.22528.Toll-like.

45. Ettinger AS, Téllez-Rojo MM, Amarasiriwardena C, et al. Levels of lead in breast milk and their relation to maternal blood and bone lead levels at one month postpartum. Environ Health Perspect. 2004;112(8):926-931. doi:10.1289/ehp.6615.

46. Téllez-Rojo MM, Hernández-Avila M, González-Cossío T, et al. Impact of breastfeeding on the mobilization of lead from bone. Am J Epidemiol. 2002;155(5):420-428. doi:10.1093/aje/155.5.420.

47. Lacasana M, Romieu I, Sanin LH, Palazuelos E, Hernandez-Avila M. Blood lead levels and calcium intake in Mexico City children under five years of age. Int J Environ Health Res. 2000;10(4):331-340. doi:10.1080/0960312002001537.

48. Romieu I, Carreon T, Lopez L, et al. Environmental urban lead exposure and blood lead levels in children of Mexico City. Environ Health Perspect. 1995;103(11):1036-1040.

49. Rothenberg SJ, Karchmer S, Schnaas L, et al. Maternal influences on cord blood lead levels. J Expo Anal Environ Epidemiol. 1996;6(2):211-227.

50. Rosado JL, López P, Kordas K, et al. Iron and/or Zinc Supplementation Did Not Reduce Blood Lead Concentrations in Children in a Randomized, Placebo-Controlled Trial. J Nutr. 2006;136:2378-2383.

51. Zhang A, Hu H, Sánchez BN, et al. Association between prenatal lead exposure and blood pressure in children. Environ Health Perspect. 2012;120(3):445-450. doi:10.1289/ehp.1103736.

52. Chaparro CM, Fornes R, Neufeld LM, Tena Alavez G, Eguía-Líz Cedillo R, Dewey KG. Early Umbilical Cord Clamping Contributes to Elevated Blood Lead Levels among Infants with Higher Lead Exposure. J Pediatr. 2007;151:506-512. doi:10.1016/j.jpeds.2007.04.056.

53. Alvear Galindo G, Carreón García J, Moreno Altamirano A, Cuéllar López JA, Yamamoto Kimura L. Lead exposure in students in Mexico City. Ecotoxicol Environ Saf. 1994;29(1):122-129. doi:10.1016/0147-6513(94)90037-X.

54. Chuang HY, Schwartz J, Gonzales-Cossio T, et al. Interrelations of lead levels in bone, venous blood, and umbilical cord blood with exogenous lead exposure through maternal plasma lead in peripartum women. Environ Health Perspect. 2001. doi:10.1289/ehp.01109527.

55. Cossío-Torres P, Calderón J, Tellez-Rojo M, Díaz-Barriga F. Factors related to health outcomes and health risk behaviors of adolescents with lead exposure. A pilot study. Salud Ment. 2013;36(1):71-79. https://www.scopus.com/inward/record.uri?eid=2-s2.0-84877021135&partnerID=40&md5=7bf5bb4ba8e2d9fd5b4c8ceb960a1b7d.

56. Meneses-González F, Richardson V, Lino-González M, Vidal MT. Niveles de plomo en sangre y factores de exposición en niños del estado de Morelos, México. Salud Publica Mex. 2003;45(SUPPL. 2):203-208. doi:10.1590/S0036-36342003000800006.

57. Ettinger AS, Téllez-Rojo MM, Amarasiriwardena C, et al. Effect of breast milk lead on infant blood lead levels at 1 month of age. Environ Health Perspect. 2004;112(14):1381-1385. doi:10.1289/ehp.6616.

58. Leal-Escalante C, Baltazar-Reyes M, Lino-González M, Palazuelos-Rendón E, Meneses-González F. Concentraciones de plomo en sangre y reprobación de escolares en la ciudad de México. Gac Med Mex. 2007;143(5):377-381.

59. Afeiche M, Peterson KE, Sánchez BN, et al. Prenatal Lead Exposure and Weight of 0-to 5-Year-Old Children in Mexico City. Environ Health Perspect. 2011;119(10):1436-1441. doi:10.1289/ehp.1003184.

60. Aguilar-Garduño C, Lacasaña M, Tellez-Rojo MM, et al. Indirect lead exposure among children of radiator repair workers. Am J Ind Med. 2003;43(6):662-667. doi:10.1002/ajim.10204.

61. Calderón-Salinas J, Valdéz-Anaya B, Mazúñiga C, Albores-Medina A. Lead exposure in a population of Mexican children. Hum Exp Toxicol. 1996;15:305-311.

62. Téllez-Rojo MM, Bellinger DC, Arroyo-Quiroz C, et al. Longitudinal associations between blood lead concentrations lower than 10 microg/dL and neurobehavioral development in environmentally exposed children in Mexico City. Pediatrics. 2006;118(2):e323-330. doi:10.1542/peds.2005-3123.

63. Azcona-Cruz MI, Rothenberg SJ, Schnaas-Arrieta L, Romero-Placeres M, Perroni-Hernández E. Niveles de plomo en sangre en niños de 8 a 10 años y su relación con la alteración en el sistema visomotor y del equilibrio. Salud Publica Mex. 2000;42(4):279-287.

64. Costa de Almeida GR, Fernandes de Freitas Tavares C, de Souza AM, et al. Whole blood, serum, and saliva lead concentrations in 6- to 8-year-old children. Sci Total Environ. 2010;408(7):1551-1556. doi:10.1016/j.scitotenv.2009.12.034.

65. Carvalho F, Silvany-Neto A, Barbosa A, Cotrim C, Tavares T. Erythrocyte protoporphyrin versus blood lead: relationship with iron status among children exposed to gross environmental pollution. Environ Res. 1995;71:11-15.

66. Costa de Almeida GR, Umbelino de Freitas C, Barbosa F, Tanus-Santos JE, Gerlach RF. Lead in saliva from lead-exposed and unexposed children. Sci Total Environ. 2009;407(5):1547-1550. doi:10.1016/j.scitotenv.2008.10.058.

67. de Carvalho Rondó PH, Carvalho M de FH, Souza MC, Moraes F. Lead, hemoglobin, zinc protoporphyrin and ferritin concentrations in children. Rev Saude Publica. 2006;40(1):71-76. doi:10.1590/S0034-89102006000100012.

68. Polido K, Olympio K, Naozuka J, et al. Association of dental enamel lead levels with risk factors for environmental exposure. Rev Saude Publica. 2010;44(5):851-858.

69. Zentner LEA, de Carvalho Rondó PH, Dias de Oliveira Latorre M do R. Blood lead concentrations in maternal and cord blood evaluated by two analytic methods. Arch Environ Occup Health. 2005;60(1):47-50. doi:10.3200/AEOH.60.1.47-50.

70. Costa de Almeida GR, de Sousa Guerra C, de Angelo Souza Leite G, et al. Lead contents in the surface enamel of primary and permanent teeth, whole blood, serum, and saliva of 6- to 8-year-old children. Sci Total Environ. 2011. doi:10.1016/j.scitotenv.2011.01.004.

71. Costa de Almeida GR, de Souza Guerra C, Tanus-Santos JE, Barbosa F, Gerlach RF. A plateau detected in lead accumulation in subsurface deciduous enamel from individuals exposed to lead may be useful to identify children and regions exposed to higher levels of lead. Environ Res. 2008;107(2):264-270. doi:10.1016/j.envres.2008.01.002.

72. Olympio KPK, Oliveira P V., Naozuka J, et al. Surface dental enamel lead levels and antisocial behavior in Brazilian adolescents. Neurotoxicol Teratol. 2010;32(2):273-279. doi:10.1016/j.ntt.2009.12.003.

73. Maleronka Ferron M, Klafke de Lima A, Nascimento Saldiva P, Gouveia N. Environmental lead poisoning among children in Porto Alegre state, Southern Brazil. Rev Saude Publica. 2012;46(2):226-233.

74. Paoliello MMB, De Capitani EM, da Cunha FG, et al. Exposure of Children to Lead and Cadmium from a Mining Area of Brazil. Environ Res. 2002;88(2):120-128. doi:10.1006/enrs.2001.4311.

75. Almeida GRC de, Pereira Saraiva M da C, Barbosa F, et al. Lead contents in the surface enamel of deciduous teeth sampled in vivo from children in uncontaminated and in lead-contaminated areas. Environ Res. 2007. doi:10.1016/j.envres.2007.03.007.

76. Gomes VE, De Sousa MDLR, Barbosa F, et al. In vivo studies on lead content of deciduous teeth superficial enamel of preschool children. Sci Total Environ. 2004;320(1):25-35. doi:10.1016/j.scitotenv.2003.08.013.

77. Oliveira da Costa Mattos RDC, Xavier EC, Domingos Mainenti HR, et al. Evaluation of calcium excretion in Brazilian infantile and young population environmentally exposed to lead. Hum Exp Toxicol. 2009;28(9):567-575. doi:10.1177/0960327109102804.

78. Arruda-Neto JDT, de Oliveira MCC, Sarkis JES, et al. Study of environmental burden of lead in children using teeth as bioindicator. Environ Int. 2009;35(3):614-618. doi:10.1016/j.envint.2008.12.005.

79. Menezes-Filho JA, Freitas de Sousa Viana G, Rodrigues Paes C. Determinants of lead exposure in children on the outskirts of Salvador, Brazil. Environ Monit Assess. 2012;184(4):2593-2603. doi:10.1007/s10661-011-2137-0.

80. de Freitas CU, De Capitani EM, Gouveia N, et al. Lead exposure in an urban community: Investigation of risk factors and assessment of the impact of lead abatement measures. Environ Res. 2007. doi:10.1016/j.envres.2006.09.004.

81. Koyashiki GAK, Paoliello MMB, Matsuo T, et al. Lead levels in milk and blood from donors to the Breast Milk Bank in Southern Brazil. Environ Res. 2010;110(3):265-271. doi:10.1016/j.envres.2009.12.001.

82. Silvany-Neto A, Carvalho F, Tavares T, et al. Lead poisoning among children of Santo Amaro, Bahia, Brazil in 1980, 1985, and 1992. Bull PAHO. 1996;30(1):51-62.

83. Counter S. Brainstem neural conduction biomarkers in lead-exposed children of Andean lead-glaze workers. J Occup Environ Med. 2002;44(9):855-864. doi:10.1097/01.jom.0000031925.77204.ca.

84. Vahter M, Counter SA, Laurell G, et al. Extensive lead exposure in children living in an area with production of lead-glazed tiles in the Ecuadorian Andes. Int Arch Occup Environ Health. 1997;70(4):282-286. doi:10.1007/s004200050220.

85. Counter SA, Buchanan LLH, Ortega F. Lead concentrations in maternal blood and breast milk and pediatric blood of Andean villagers: 2006 follow-up investigation. J Occup Environ Med. 2007;49(3):302-309. doi:10.1097/jom.0b013e31803225b0.

86. Counter SA, Buchanan LH, Ortega F, Laurell G. Normal auditory brainstem and cochlear function in extreme pediatric plumbism. J Neurol Sci. 1997;152(1):85-92. doi:10.1016/S0022-510X(97)00149-4.

87. Counter S, Buchanan L, Laurell G, Ortega F. Field screening or blood lead levels in a remote Andean villages. Neurotoxicology. 1998;19(6):871-878.

88. Counter SA, Buchanan LH, Ortega F. Zinc protoporphyrin levels, blood lead levels and neurocognitive deficits in Andean children with chronic lead exposure. Clin Biochem. 2008;41(1-2):41-47. doi:10.1016/j.clinbiochem.2007.10.002.

89. Counter SA, Buchanan LH, Ortega F, Rifai N, Shannon MW. Comparative analysis of zinc protoporphyrin and blood lead levels in lead-exposed Andean children. Clin Biochem. 2007;40(11):787-792. doi:10.1016/j.clinbiochem.2007.03.003.

90. Counter SA, Buchanan LH, Ortega F. Neurocognitive Impairment in Lead-Exposed Children of Andean Lead-Glazing Workers. J Occup Environ Med. 2005;47(3):306-312. doi:http://dx.doi.org/10.1097/01.jom.0000155717.45594.65.

91. Counter SA, Vahter M, Laurell G, Buchanan LH, Ortega F, Skerfving S. High lead exposure and auditory sensory-neural function in Andean children. Environ Health Perspect. 1997;105(5):522-526.

92. Counter SA, Buchanan LH, Ortega F. Current pediatric and maternal lead levels in blood and breast milk in Andean inhabitants of a lead-glazing enclave. J Occup Environ Med. 2004;46(9):967-973. doi:10.1097/01.jom.0000137712.21963.76.

93. Buchanan LLH, Counter SAS, Ortega F. Environmental lead exposure and otoacoustic emissions in Andean children. J Toxicol Environ Health. 2011;74(19):1280-1293. doi:10.1002/ana.22528.Toll-like.

94. Lozoff B, Jimenez E, Wolf AW, et al. Higher Infant Blood Lead Levels with Longer Duration of Breastfeeding. J Pediatr. 2009;155(5):663-667. doi:10.1016/j.jpeds.2009.04.032.

95. Sepúlveda V, Vega J, Delgado I. Exposición severa a plomo ambiental en una población infantil de Antofagasta, Chile. Rev Med Chil. 2000;128(2):221-232.

96. Sánchez-Cortez J, Ilabaca-Marileo M, Martín M, Viñas M, Bravo-Méndez R. Prevalence of lead in blood in scholar children in Santiago of Chile. Salud Publica Mex. 2003;45(sup 2):S264-S268.

97. Frenz P, Vega J, Marchetti N, et al. Chronic exposure to environmental lead in Chilean infants. Rev Med Chil. 1997;125:1137-1144.

98. Pino P, Walter T, Oyarzún MJ, Burden MJ, Lozoff B. Rapid drop in infant blood lead levels during the transition to unleaded gasoline use in Santiago, Chile. Arch Environ Health. 2004;59(4):182-187. doi:10.3200/AEOH.59.4.182-187.

99. Coria C, Cabello A, Tassara E, et al. Efectos clínicos a largo plazo en niños intoxicados con plomo en una región del sur de Chile. Rev Med Chil. 2009;137(8):1037-1044. doi:10.4067/S0034-98872009000800006.

100. Pérez-Bravo F, Ruz M, Morán-Jiménez M, et al. Association between aminolevulinate dehydrase genotypes and blood lead levels in children from a lead-contaminated area in Antofagasta, Chile. Arch Environ Contam Toxicol. 2004;47:276-280. doi:10.1007/s00244-004-2215-1.

101. Iglesias V, Steenland K, Maisonet M, Pino P. Exposure to lead from a storage site associated with intellectual impairment in chilean children living nearby. Int J Occup Environ Health. 2011;17(4):314-321. doi:10.1179/107735211799041841.

102. Kordas K, Ardoino G, Ciccariello D, et al. Association of maternal and child blood lead and hemoglobin levels with maternal perceptions of parenting their young children. Neurotoxicology. 2011. doi:10.1016/j.neuro.2011.09.001.

103. Cousillas A, Pereira L, Heller T, Alvarez C, Mañay N. Impacts of multidisciplinary actions on environmental lead exposure in Uruguay. Environ Geochem Health. 2012;34(2):207-211. doi:10.1007/s10653-011-9426-y.

104. Queirolo EI, Ettinger AS, Stoltzfus RJ, et al. Association of anemia, child and family characteristics with elevated blood lead concentrations in preschool children from Montevideo, Uruguay. Arch Environ Occup Health. 2010;65(2):94-100. doi:10.1080/19338240903390313.

105. Cousillas A, Pereira L, Alvarez C, et al. Comparative study of blood lead levels in Uruguayan children (1994-2004). Biol Trace Elem Res. 2008;122:19-25.

106. Schütz A, Barregård L, Sällsten G, et al. Blood lead in Uruguayan children and possible sources of exposure. Environ Res. 1997;74:17-23. doi:10.1006/enrs.1997.3742.

107. Mañay N, Alonzo C, Dol I. Contaminación por plomo en el barrio La Teja: Montevideo, Uruguay. Salud Publica Mex. 2003;45(2):252-278.

108. Anticona C, Bergdahl IA, Lundh T, Alegre Y, San Sebastian M. Lead exposure in indigenous communities of the Amazon basin, Peru. Int J Hyg Environ Health. 2011;215(1):59-63. doi:10.1016/j.ijheh.2011.07.003.

109. Vega-Dienstmaier JM, Salinas-Piélago JE, Gutiérrez-Campos MDR, et al. Lead levels and cognitive abilities in Peruvian children. Rev Bras Psiquiatr. 2006;28(1):33-39. doi:10.1590/S1516-44462006000100008.

110. Espinoza R, Hernández-Avila M, Narciso J, et al. Determinants of blood-lead levels in children in Callao and Lima metropolitan area. Salud Publica Mex. 2003;45(SUPPL. 2):209-219. doi:10.1590/S0036-36342003000800007.

111. Naeher LP, Rubin CS, Hernandez-Avila M, et al. Use of isotope ratios to identify sources contributing to pediatric lead poisoning in Peru. Arch Environ Health. 2003;58(9):579-589. http://www.ncbi.nlm.nih.gov/pubmed/15369276.

112. Anticona C, Bergdahl IA, San Sebastian M. Lead exposure among children from native communities of the Peruvian Amazon basin. Rev Panam Salud Publica. 2012;31(4):296-302. doi:dx.doi.org/S1020-49892012000400005.

113. Olivero-Verbel J, Duarte D, Echenique M, Guette J, Johnson-Restrepo B, Parsons PJ. Blood lead levels in children aged 5-9 years living in Cartagena, Colombia. Sci Total Environ. 2007;372(2-3):707-716. doi:10.1016/j.scitotenv.2006.10.025.

114. Filigrana PA, Méndez F. Blood lead levels in schoolchildren living near an industrial zone in Cali, Colombia: The role of socioeconomic condition. Biol Trace Elem Res. 2012. doi:10.1007/s12011-012-9429-20.

115. Hurtado CM, Gutiérrez M, Echeverry J. Aspectos clínicos y niveles de plomo en niños expuestos de manera paraocupacional en el proceso de reciclaje de baterías de automóviles en las localidades de Soacha y Bogotá, D. C. Biomédica. 2008;2828:116-125.

116. Gardner J, Walker S, Chang S, Vutchkov M, Lalor G. Undernutrition and elevated blood lead levels: effects on psychomotor development among Jamaican children. Public Health Nutr. 1998;1(3):177-179. http://ovidsp.ovid.com/ovidweb.cgi?T=JS&PAGE=reference&D=med4&NEWS=N&AN=10933415.

117. Lalor G, Vutchkov M, Bryan S. Blood lead levels of Jamaican children island-wide. Sci Total Environ. 2007;374(2-3):235-241. doi:10.1016/j.scitotenv.2006.12.045.

118. Lalor GC, Vutchkov MK, Bryan ST, et al. Acute lead poisoning associated with backyard lead smelting in Jamaica. West Indian Med J. 2006;55(6):394-398. doi:10.1590/S0043-31442006000600005.

119. Disalvo L, Aab C, Pereyras S, et al. Blood lead levels in children from the city of La Plata, Argentina. Relationship with iron deficiency and lead exposure risk factors. Arch Argent Pediatr. 2009;107(4):300-306.

120. Martínez S, Simonella L, Hansen C, Rivolta S, Cancela L, Virgolini M. Blood lead levels and enzymatic biomarkers of environmental lead exposure in children in Córdoba, Argentina, after the ban of leaded gasoline. Hum Exp Toxicol. 2013;32(5):449-463. doi:10.1177/0960327112454893.

121. Espinosa C, Rojas M, Seijas D. El sistema geográfico de información y las concentraciones de plomo en sangre en una población infantil venezolana. Salud Publica Mex. 2006;48(2):84-93.

122. Rojas M, Espinosa C, Seijas D. Asociación entre plomo en sangre y parámetros sociodemográficos en población infantil. Rev Saude Publica. 2003;37(4):503-509. www.fsp.usp.br/rsp.

123. Charalambous A, Demoliou K, Mendez M, Coye R, Solorzano G, Papanastasiou E. Screening for lead exposure in children in Belize. Rev Panam Salud Pública. 2009;25(1):47-50. http://www.ncbi.nlm.nih.gov/pubmed/19341523.

124. Ruiz-Castell M, Paco P, Barbieri FL, et al. Child neurodevelopment in a Bolivian mining city. Environ Res. 2012;112:147-154. doi:10.1016/j.envres.2011.12.001.

125. Kaul B, Sandhu RS, Depratt C, Reyes F. Follow-up screening of lead-poisoned children near an auto battery recycling plant, Haina, Dominican Republic. Environ Health Perspect. 1999;107(11):917-920. doi:10.2307/3454481.

126. Simon Rajkumar W, Manohar J, Doon R, et al. Blood lead levels in primary school children in Trinidad and Tobago. Sci Total Environ. 2006;361(1-3):81-87. doi:10.1016/j.scitotenv.2005.05.012.

127. Yáñez L, García-Nieto E, Rojas E, et al. DNA damage in blood cells from children exposed to arsenic and lead in a mining area. Environ Res. 2003;93(3):231-240. doi:10.1016/j.envres.2003.07.005.

128. Roy A, Kordas K, Lopez P, et al. Association between arsenic exposure and behavior among first-graders from Torreón, Mexico. Environ Res. 2011;111(5):670-676. doi:10.1016/j.envres.2011.03.003.

129. Méndez-Gómez J, García-Vargas GG, López-Carrillo L, et al. Genotoxic effects of environmental exposure to arsenic and lead on children in Region Lagunera, Mexico. Ann N Y Acad Sci. 2008;1140:358-367. doi:10.1196/annals.1454.027.

130. Wyatt CJ, Lopez Quiroga V, Olivas Acosta RT, Méndez RO. Excretion of Arsenic (As) in Urine of Children, 7–11 Years, Exposed to Elevated Levels of As in the City Water Supply in Hermosillo, Sonora, Meico. Environ Res. 1998;78:19-24.

131. Luna AL, Acosta-Saavedra LC, Lopez-Carrillo L, et al. Arsenic alters monocyte superoxide anion and nitric oxide production in environmentally exposed children. Toxicol Appl Pharmacol. 2010;245(2):244-251. doi:10.1016/j.taap.2010.03.006.

132. Sampayo-Reyes A, Hernández A, El-Yamani N, et al. Arsenic induces DNA damage in environmentally exposed Mexican children and adults. Influence of GSTO1 and AS3MT polymorphisms. Toxicol Sci. 2010;117(1):63-71. doi:10.1093/toxsci/kfq173.

133. Meza MM, Yu L, Rodriguez YY, et al. Developmentally restricted genetic determinants of human arsenic metabolism: association between urinary methylated arsenic and CYT19 polymorphisms in children. Environ Health Perspect. 2005;113(6):775-781. doi:10.1289/ehp.7780.

134. Carrizales L, Razo I, Téllez-Hernández JI, et al. Exposure to arsenic and lead of children living near a copper-smelter in San Luis Potosi, Mexico: Importance of soil contamination for exposure of children. Environ Res. 2006;101(1):1-10. doi:10.1016/j.envres.2005.07.010.

135. Calderón J, Navarro M, Jimenez-Capdeville M, et al. Exposure to Arsenic and Lead and Neuropsychological Development in Mexican Children. Environ Res. 2001;85(2):69-76. doi:10.1006/enrs.2000.4106.

136. Gamiño-Gutiérrez SP, González-Pérez CI, Gonsebatt ME, Monroy-Fernández MG. Arsenic and lead contamination in urban soils of Villa de la Paz (Mexico) affected by historical mine wastes and its effect on children's health studied by micronucleated exfoliated cells assay. Environ Geochem Health. 2013;35(1):37-51. doi:10.1007/s10653-012-9469-8.

137. Pineda-Zavaleta AP, García-Vargas G, Borja-Aburto VH, et al. Nitric oxide and superoxide anion production in monocytes from children exposed to arsenic and lead in region Lagunera, Mexico. Toxicol Appl Pharmacol. 2004;198(3):283-290. doi:10.1016/j.taap.2003.10.034.

138. Rocha-Amador DO, Calderón J, Carrizales L, Costilla-Salazar R, Pérez-Maldonado IN. Apoptosis of peripheral blood mononuclear cells in children exposed to arsenic and fluoride. Environ Toxicol Pharmacol. 2011;32(3):399-405. doi:10.1016/j.etap.2011.08.004.

139. Rosado JL, Ronquillo D, Kordas K, et al. Arsenic exposure and cognitive performance in Mexican Schoolchildren. Environ Health Perspect. 2007;115(9):1371-1375. doi:10.1289/ehp.9961.

140. Monroy-Torres R, Macías AE, Gallaga-Solorzano JC, Santiago-García EJ, Hernandez I. Arsenic in mexican children exposed to contaminated well water. Ecol Food Nutr. 2009;48(1):59-75. doi:10.1080/03670240802575519.

141. Jasso-Pineda Y, Espinosa-Reyes G, González-Mille D, et al. An integrated health risk assessment approach to the study of mining sites contaminated with arsenic and lead. Integr Environ Assess Manag. 2007;3(3):344-350. http://www.ncbi.nlm.nih.gov/pubmed/17695107.

142. Osorio-Yáñez C, Ayllon-Vergara JC, Aguilar-Madrid G, et al. Carotid intima-media thickness and plasma asymmetric dimethylarginine in Mexican children exposed to inorganic arsenic. Environ Health Perspect. 2013;121(9):1090-1096. doi:10.1289/ehp.1205994.

143. Jasso-Pineda Y, Díaz-Barriga F, Calderón J, Yáñez L, Carrizales L, Pérez-Maldonado IN. DNA damage and decreased DNA repair in peripheral blood mononuclear cells in individuals exposed to arsenic and lead in a mining site. Biol Trace Elem Res. 2012. doi:10.1007/s12011-011-9237-0.

144. Rager JE, Bailey KA, Smeester L, et al. Prenatal arsenic exposure and the epigenome: Altered microRNAs associated with innate and adaptive immune signaling in newborn cord blood. Environ Mol Mutagen. 2014. doi:10.1002/em.21842.

145. Rocha-Amador D, Navarro ME, Carrizales L, Morales R, Calderón J. Decreased intelligence in children and exposure to fluoride and arsenic in drinking water. Cad Saude Publica. 2007;23(sup 4):S579-S587. doi:10.1590/S0102-311X2007001600018.

146. Grijalva-Haro MI, Barba-Leyva ME, Laborín-Alvarez A. Ingestión y excreción de fluoruros en niños de Hermosillo, Sonora, México. Salud Publica Mex. 2001;43(2):127-134. doi:10.1590/S0036-36342001000200008.

147. Juárez-López MLA, Hernández-Guerrero JC, Jiménez-Farfán D, Molina-Frechero N, Murrieta-Pruneda F, López-Jiménez G. Excreción urinaria de flúor por preescolares en la ciudad de México. Rev Investig Clin. 2008;60(3):241-247.

148. Molina-Frechero N, Pierdant-Rodriguez A, Oropeza-Oropeza A, Bologna-Molina R. Fluorosis and dental caries: an assessment of risk factors in Mexican children. Rev Investig Clin. 2012;64(1):67-73. http://www.ncbi.nlm.nih.gov/pubmed/22690531.

149. García-Pérez A, Irigoyen-Camacho ME, Borges-Yáñez A. Fluorosis and dental caries in mexican schoolchildren residing in areas with different water fluoride concentrations and receiving fluoridated salt. Caries Res. 2013;47(4):299-308. doi:10.1159/000346616.

150. Chacón LFG, López MLAJ, Frechero NM. Prevalencia de fluorosis dental y consumo de fluoruros ocultos en escolares del municipio de Nezahualcóyotl. Gac Med Mex. 2009;145(4):263-267.

151. Beltrán-Valladares PR, Cocom-Tun H, Casanova-Rosado JF, Vallejos-Sánchez AA, Medina-Solís CE, Maupomé G. Prevalencia de fluorosis dental y fuentes adicionales de exposición a

fluoruro como factores de riesgo a fluorosis dental en escolares de Campeche, México. Rev Investig Clin. 2005;57(4):532-539.

152. Vallejos-Sánchez AA, Medina-Solís CE, Casanova-Rosado JF, Maupomé G, Casanova-Rosado AJ, Minaya-Sánchez M. Defectos del esmalte, caries en dentición primaria, fuentes de fluoruro y su relación con caries en dientes permanentes. Gac Sanit. 2007;21(3):227-234. doi:10.1157/13106806.

153. Grimaldo M, Borja-Aburto VH, Ramírez AL, Ponce M, Rosas M, Díaz-Barriga F. Endemic fluorosis in San Luis Potosi, Mexico I. Identification of risk factors associated with human exposure to fluoride. Environ Res. 1995;68:25-30.

154. Pontiago-Loyola AP, Medina-Solis CE, Borges-Yañez SA, Patiño-Marín N, Islas-Márquez A, Maupome G. Prevalence and severity of dental caries in adolescents aged 12 and 15 living in communities with various fluoride concentrations. J Public Health Dent. 2007;67(1):8-13.

155. Martínez-Mier EA, Soto-Rojas AE, Ureña-Cirett JL, Stookey GK, Dunipace AJ. Fluoride intake from foods, beverages and dentifrice by children in Mexico. Community Dent Oral Epidemiol. 2003;31(3):221-230. doi:10.1034/j.1600-0528.2003.00043.x.

156. Torres-Agustín R, Rodríguez-Agudelo Y, Schilmann A, et al. Effect of environmental manganese exposure on verbal learning and memory in Mexican children. Environ Res. 2013;121:39-44. doi:10.1016/j.envres.2012.10.007.

157. Riojas-Rodríguez H, Solís-Vivanco R, Schilmann A, et al. Intellectual function in Mexican children living in a mining area and environmentally exposed to manganese. Environ Health Perspect. 2010;118(10):1465-1470. doi:10.1289/ehp.0901229.

158. Henn BC, Ettinger AS, Schwartz J, et al. Early Postnatal Blood Manganese Levels and Children's Neurodevelopment. Epidemiology. 2010;21(4):433-439. doi:10.1097/EDE.0b013e3181df8e52.

159. Montes S, Schilmann A, Riojas-Rodriguez H, et al. Serum prolactin rises in Mexican school children exposed to airborne manganese. Environ Res. 2011;111(8):1302-1308. doi:10.1016/j.envres.2011.09.015.

160. Hernández-Bonilla D, Schilmann A, Montes S, et al. Environmental exposure to manganese and motor function of children in Mexico. Neurotoxicology. 2011;32(5):615-621. doi:10.1016/j.neuro.2011.07.010.

161. Moreno ME, Acosta-Saavedra LC, Meza-Figueroa D, et al. Biomonitoring of metal in children living in a mine tailings zone in Southern Mexico: A pilot study. Int J Hyg Environ Health. 2010;213(4):252-258. doi:10.1016/j.ijheh.2010.03.005.

162. Carrillo-Ponce MDL, Martínez-Ordaz VA, Velasco-Rodríguez VM, Hernández-García A, Hernández-Serrano MC, Sanmiguel F. Serum lead, cadmium, and zinc levels in newborns with neural tube defects from a polluted zone in Mexico. Reprod Toxicol. 2004;19(2):149-154. doi:10.1016/j.reprotox.2004.07.003.

163. Galicia-Garcia V, Rojas-L6pez M, Rojas ' R, et al. Cadmium levels in maternal, cord and newborn blood in Mexico city. Toxicol Lett. 1997;91:57-61. http://www.sciencedirect.com/science/article/pii/S0378427497038691.

164. Sakuma AM, Mello De Capitani E, Ribeiro Figueiredo B, et al. Arsenic exposure assessment of children living in a lead mining area in Southeastern Brazil. Cad Saude Publica. 2010;26(2):391-398.

165. Castro Martins C, Oliveira MJ, Almeida Pordeus I, Cury JA, Martins Paiva S. Association between socioeconomic factors and the choice of dentifrice and fluoride intake by children. Int J Environ Res Public Health. 2011;8(11):4284-4299. doi:10.3390/ijerph8114284.

166. Ribeiro do Nascimento HA, Soares Ferreira JM, Granville-Garcia AF, Melo de Brito Costa EM, Almeida Cavalcante AL, Correia Sampaio F. Estimation of Toothpaste Fluoride Intake in Preschool Children. Braz Dent J. 2013;24(2):142-146. doi:10.1590/0103-6440201302087.

167. Fonseca Cortes D, Ellwood R, O'Mullane D, de Magalhaes Bastos J. Drinking water fluoride levels, dental fluorosis, and caries experience in Brazil. J Public Health Dent. 1996;56(4):226-228.

168. Borges Miziara AP, Tucunduva Philippi S, Mauad Levy F, Rabelo Buzalaf MA. Fluoride ingestion from food items and dentifrice in 2-6-year-old Brazilian children living in a fluoridated area using a semiquantitative food frequency questionnaire. Community Dent Oral Epidemiol. 2009;37(4):305-315. doi:10.1111/j.1600-0528.2009.00477.x.

169. Forte FDS, Moimaz SAS, Sampaio FC. Urinary fluoride excretion in children exposed to fluoride toothpaste and to different water fluoride levels in a tropical area of Brazil. Braz Dent J. 2008. doi:10.1590/S0103-64402008000300007.

170. Carvalho TS, Kehrle HM, Sampaio FC. Prevalence and severity of dental fluorosis among students from João Pessoa, PB, Brazil. Braz Oral Res. 2007;21(3):198-203. doi:10.1590/S1806-83242007000300002.

171. Dini E, Holt R, Bedi R. Prevalence of caries and developmental defects of enamel in 9-10 year old children living in areas in Brazil with differing water fluoride histories. Br Dent J. 2000;188(3):146-149.

172. Correia Sampaio F, Nasir Mohammad Nazmul Hossain A, Ramm von der Fehr F, Arneberg P, And B. Dental caries and sugar intake of children from rural areas with different water fluoride levels. Community Dent Oral Epidemiol. 2000;28:307-313.

173. Hashizume LN, Mathias TC, Cibils DM, Maltz M. Effect of the widespread use of fluorides on the occurrence of hidden caries in children. Int J Pediatr Dent. 2013;23(1):72-76. doi:10.1111/j.1365-263X.2012.01231.x.

174. Omena LMF, Silva MF de A, Pinheiro CC, Cavalcante JC, Correia Sampaio F. Fluoride intake from drinking water and dentifrice by children living in a tropical area of Brazil. J Appl Oral Sci. 2006;14(5):382-387. http://www.scielo.br/scielo.php?script=sci_arttext&pid=S1678-77572006000500015&lng=pt&nrm=iso&tlng=en.

175. Feldens C, Kuchenbecker Rösing C, Zimmermann dos Santos B, Rodríguez Cordeiro M. Pattern of fluoride-containing dentifrice use and associated factors in preschool children from Ijuí, South Brazil. Oral Heal Prev Dent. 2010;8(3):277-285.

176. Ferreira EF, Vargas AMD, Castilho LS, Velásquez LNM, Fantinel LM, Abreu MHN. Factors associated to endemic dental fluorosis in Brazilian rural communities. Int J Environ Res Public Health. 2010;7(8):3115-3128. doi:10.3390/ijerph7083115.

177. Menezes-Filho JA, Novaes C de O, Moreira JC, Sarcinelli PN, Mergler D. Elevated manganese and cognitive performance in school-aged children and their mothers. Environ Res. 2011;111(1):156-163. doi:10.1016/j.envres.2010.09.006.

178. Farias AC, Cunha A, Benko CR, et al. Manganese in Children with Attention-Deficit/Hyperactivity Disorder: Relationship with Methylphenidate Exposure. J Child Adolesc Psychopharmacol. 2010;20(2):113-118. doi:10.1089/cap.2009.0073.

179. Menezes-Filho JA, Paes CR, Ángela ÁM, Moreira JC, Sarcinelli PN, Mergler D. High levels of hair manganese in children living in the vicinity of a ferro-manganese alloy production plant. Neurotoxicology. 2009;30(6):1207-1213. doi:10.1016/j.neuro.2009.04.005.

180. Bocayuva Tavares LM, Câmara VM, Malm O, de Oliveira Santos EC. Performance on neurological development tests by riverine children with moderate mercury exposure in Amazonia, Brazil. Cad Saude Publica. 2005;21(4):1160-1167. doi:10.1590/S0102-311X2005000400018.

181. Marques RC, Dórea JG, Leão RS, et al. Role of methylmercury exposure (from Fish Consumption) on growth and neurodevelopment of children under 5 years of age living in a transitioning (tin-mining) area of the Western Amazon, Brazil. Arch Environ Contam Toxicol. 2012;62(2):341-350. doi:10.1007/s00244-011-9697-4.

182. Marques RC, Dórea JG, Bastos WR, Malm O. Changes in children hair-Hg concentrations during the first 5 years: Maternal, environmental and iatrogenic modifying factors. Regul Toxicol Pharmacol. 2007. doi:10.1016/j.yrtph.2007.05.001.

183. Marques RC, Dórea JG, McManus C, et al. Hydroelectric reservoir inundation (Rio Madeira Basin, Amazon) and changes in traditional lifestyle: impact on growth and neurodevelopment of pre-school children. Public Health Nutr. 2011;14(4):661-669. doi:10.1017/S136898001000248X.

184. Chevrier C, Sullivan K, White RF, Comtois C, Cordier S, Grandjean P. Qualitative assessment of visuospatial errors in mercury-exposed Amazonian children. Neurotoxicology. 2009;30(1):37-46. doi:10.1016/j.neuro.2008.09.012.

185. Fonseca M de F, Dórea JG, Bastos WR, Marques RC, Torres JPM, Malm O. Poor psychometric scores of children living in isolated riverine and agrarian communities and fish-methylmercury exposure. Neurotoxicology. 2008;29(6):1008-1015. doi:10.1016/j.neuro.2008.07.001.

186. Dórea JG, Marques RC, Isejima C. Neurodevelopment of Amazonian infants: Antenatal and postnatal exposure to methyl- and ethylmercury. J Biomed Biotechnol. 2012;2012. doi:10.1155/2012/132876.

187. Barbosa AC, Silva SRL, Dórea JG. Concentration of mercury in hair of indigenous mothers and infants from the Amazon basin. Arch Environ Contam Toxicol. 1998;34(1):100-105. doi:10.1007/s002449900291.

188. Boischio AAP, Henshel DS. Linear Regression Models of Methyl Mercury Exposure during Prenatal and Early Postnatal Life among Riverside People along the Upper Madeira River, Amazon. Environ Res. 2000;83(2):150-161. doi:10.1006/enrs.2000.4050.

189. Malm O, Dórea JG, Barbosa AC, Pinto FN, Weihe P. Sequential hair mercury in mothers and children from a traditional riverine population of the Rio Tapajós, Amazonia: Seasonal changes. Environ Res. 2010;110(7):705-709. doi:10.1016/j.envres.2010.07.008.

190. Simões Dutra MD, Maura de Jesus I, de Oliveira Santos EC, et al. Longitudinal assessment of mercury exposure in schoolchildren in an urban area of the Brazilian Amazon. Cad Saude Publica. 2012;28(8):1539-1545. doi:10.1590/S0102-311X2012000800012.

191. de Olveira Santos E, Maura de Jesus I, da Silva Brabo E, et al. Mercury exposures in riverside Amazon communities in Pará, Brazil. Environ Res. 2000;84:100-107. doi:10.1006/enrs.2000.4088.

192. Dórea JG, Marques RC, Abreu L. Milestone achievement and neurodevelopment of rural Amazonian toddlers (12 to 24 months) with different methylmercury and ethylmercury exposure. J Toxicol Environ Health. 2014;77:1-13.

193. Dórea JG, Barbosa AC, Ferrari Í, de Souza JR. Fish consumption (hair mercury) and nutritional status of Amazonian Amer-Indian children. Am J Hum Biol. 2005;17(4):507-514. doi:10.1002/ajhb.20410.

194. Marques RC, Garrofe Dórea J, Rodrigues Bastos W, de Freitas Rebelo M, de Freitas Fonseca M, Malm O. Maternal mercury exposure and neuro-motor development in breastfed infants from Porto Velho (Amazon), Brazil. Int J Hyg Environ Health. 2007;210(1):51-60. doi:10.1016/j.ijheh.2006.08.001.

195. Pinheiro MCN, Crespo-López MEM, Vieira JLF, et al. Mercury pollution and childhood in Amazon riverside villages. Environ Int. 2007;33(1):56-61. doi:10.1016/j.envint.2006.06.024.

196. Carneiro MFH, Rhoden CR, Amantéa SL, Barbosa F. Low concentrations of selenium and zinc in nails are associated with childhood asthma. Biol Trace Elem Res. 2011;144(1-3):244-252. doi:10.1007/s12011-011-9080-3.

197. Carneiro MFH, Grotto D, Batista BL, Rhoden CR, Barbosa F. Background values for essential and toxic elements in children's nails and correlation with hair levels. Biol Trace Elem Res. 2011;144(1-3):339-350. doi:10.1007/s12011-011-9102-1.

198. Silverio Amancio O, Alves Chaud D, Yanaguibashi G, Esteves Hilário M. Copper and zinc intake and serum levels in patients with juvenile rheumatoid arthritis. Eur J Clin Nutr. 2003;57(5):706-712. doi:10.1038/sj.ejcn.1601601.

199. Barretto JR, Silva LR, Leite ME, et al. Poor zinc and selenium status in phenylketonuric children and adolescents in Brazil. Nutr Res. 2008;28(3):208-211. doi:10.1016/j.nutres.2007.12.009.

200. Garcia Pantuzo MC, Gonçalves Zenóbio E, de Andrade Marigo H, Fernandes Zenóbio MA. Hypersensitivity to conventional and to nickel-free orthodontic brackets. Braz Oral Res. 2007;21(4):298-302. doi:10.1590/S1806-83242007000400003.

201. Hopenhayn-Rich C, Browning SR, Hertz-Picciotto I, Ferreccio C, Peralta C, Gibb H. Chronic arsenic exposure and risk of infant mortality in two areas of Chile. Environ Health Perspect. 2000;108(7):667-673. doi:10.2307/3434889.

202. Liaw J, Marshall G, Yuan Y, Ferreccio C, Steinmaus C, Smith AH. Increased childhood liver cancer mortality and arsenic in drinking water in northern Chile. Cancer Epidemiol Biomarkers Prev. 2008;17(8):1982-1987. doi:10.1158/1055-9965.EPI-07-2816.

203. Linossier A, Carvajal P, Donoso E, Orrego M. Fluorosis dental: recuento de Streptococcus mutans en escolares provenientes de la Primera Región de Chile. Estudio longitudinal. Rev Med Chil. 1999;127(12):1462-1468.

204. Mella S, Molina X, Atalah E. Prevalencia de fluorosis dental endémica en relación al contenido de fluoruros en las aguas de abasto público. Rev Med Chil. 1994;122:1263-1270.

205. Villa A, Cabezas L, Anabalón M, Garza E. The fractional urinary fluoride excretion of adolescents and adults under customary fluoride intake conditions, in a community with 0.6 mg F/L in its drinking water. Community Dent Health. 2004;21:11-18.

206. Villa AE, Guerrero S, Icaza G, Villalobos J, Anabaló N M. Dental fluorosis in Chilean caries in Chilean children in order to establish the most appropriate water fluo- ridation level in Chile. Community Dent Oral Epidemiol. 1998;26(26):310-315.

207. Mariño R, Villa A, Weitz A, Guerrero S. Prevalence of fluorosis in children aged 6-9 years-old who participated in a milk fluoridation programme in Codegua, Chile. Community Dent Health. 2004;21(2):143-148.

208. Villa AE, Guerrero S, Villalobos J. Estimation of optimal concentration of fluoride in drinking water under conditions prevailing in Chile. Community Dent Oral Epidemiol. 1998;26(26):249-255. doi:10.1111/j.1600-0528.1998.tb01958.x.

209. Iglesias A V, Burgos D S, Marchetti P N, Silva Z C, Pino Zúñiga P. Nivel de níquel urinario en niños residentes en ciudades cercanas a megafuentes. Rev Med Chil. 2008;136(8):1039-1046. doi:10.4067/S0034-98872008000800013.

210. Counter SA, Buchanan LH, Ortega F, Counter A, Buchanan LH, Ortega F. Neurocognitive Screening of Mercury-exposed children of Andean gold miners. Int J Occup Environ Health. 2006;12:209-214.

211. Counter SA, Buchanan LH, Ortega F. Mercury levels in urine and hair of children in an Andean gold-mining settlement. Int J Occup Environ Health. 2005;11(2):132-137. doi:10.1179/oeh.2005.11.2.132.

212. Counter S. Neurophysiological anomalies in brainstem responses of Mercury-exposed children of Andean gold miners. J Occup Environ Med. 2003;45(1):87-95. doi:10.1097/01.jom.0000048165.87707.fd.

213. Counter SA, Buchanan LH, Ortega F, Laurell G. Elevated blood mercury and neuro-otological observations in children of the ecuadorian gold mines. J Toxicol Environ Heal - Part A. 2002;65(2):149-163. doi:10.1080/152873902753396785.

214. Counter S, Buchanan L, Laurell G, Ortega F. Blood mercuryand auditory neuro-sensory responses in children and adults in the Nambija gold mining area of Ecuador. Neurotoxicology. 1998;19(2):185-196.

215. Hrubá F, Strömberg U, Černá M, et al. Blood cadmium, mercury, and lead in children: An international comparison of cities in six European countries, and China, Ecuador, and Morocco. Environ Int. 2012. doi:10.1016/j.envint.2011.12.001.

216. Montero M, Rojas-Sanchez F, Socorro M, Torres J, Acevedo AM. Experiencia de caries y fluorosis dental en escolares que consumen agua con diferentes concentraciones de fluoruro en Maiquetía, Estado Vargas, Venezuela. Invest Clin. 2007;48(1):5-19.

217. Rojas M, Seijas D, Agreda O, Rodríguez M. Biological monitoring of mercury exposure in individuals referred to a toxicological center in Venezuela. Sci Total Environ. 2006. doi:10.1016/j.scitotenv.2005.01.023.

218. Loui A, Raab A, Braetter P, Obladen M, De Braetter V. Selenium status in term and preterm infants during the first months of life. Eur J Clin Nutr. 2008;62(3):349-355. doi:10.1038/sj.ejcn.1602715.

219. Brunetto M, Alarcón O, Dávila E, et al. Serum trace elements and fat-soluble vitamins A and E in healthy pre-school children from a Venezuelan rural community. J Trace Elem Med Biol. 1999;13(1-2):40-50. doi:10.1016/S0946-672X(99)80022-4.

220. Alarcón O, Reinosa Fuller J, Silva T, et al. Serum level of Zn Cu and Fe in healthy schoolchildren residing in Mérida, Venezuela. Arch Latinoam Nutr. 1997;47(2):118-122.

221. Benefice E, Luna Monrroy SJ, Lopez Rodriguez RW. A nutritional dilemma: Fish consumption, mercury exposure and growth of children in Amazonian Bolivia. Int J Environ Health Res. 2008;18(6):415-427. doi:10.1080/09603120802272235.

222. Luna Monrroy S, Lopez R, Roulet M, Benefice E. Lifestyle and mercury contamination of Amerindian populations along the Beni River (lowland Bolivia). J Environ Health. 2008;71(4):44-50.

223. Stassen MJM, Preeker NL, Ragas AMJ, van de Ven MWPM, Smolders AJP, Roeleveld N. Metal exposure and reproductive disorders in indigenous communities living along the Pilcomayo River, Bolivia. Sci Total Environ. 2012;427-428:26-34. doi:10.1016/j.scitotenv.2012.03.072.

224. Weisstaub G, Medina M, Pizarro F, Araya M. Copper, Iron, and Zinc Status in Children with Moderate and Severe Acute Malnutrition Recovered Following WHO Protocols. Biol Trace Elem Res. 2008;124:1-11. doi:10.1007/s12011-008-8090-2.

225. Rahbar MH, Samms-Vaughan M, Ardjomand-Hessabi M, et al. The role of drinking water sources, consumption of vegetables and seafood in relation to blood arsenic concentrations of Jamaican children with and without Autism Spectrum Disorders. Sci Total Environ. 2012;433:362-370. doi:10.1016/j.scitotenv.2012.06.085.

226. Warpeha R, Marthaler T. Urinary fluoride excretion in Jamaica in relation to fluorinated salt. Caries Res. 1995;29:35-41.

227. Rahbar MH, Samms-Vaughan M, Loveland KA, et al. Seafood consumption and blood mercury concentrations in Jamaican children with and without autism spectrum disorder. Neurotox Res. 2013;23(1):22-38. doi:10.1007/s12640-012-9321-z.Seafood.

228. Wickre JB, Folt CL, Sturup S, Karagas MR. Environmental exposure and fingernail analysis of arsenic and mercury in children and adults in a Nicaraguan gold mining community. Arch Environ Health. 2004;59(8):400-409.

229. Saunders JE, Jastrzembski BG, Buckey JC, Enriquez D, Mackenzie TA, Karagas MR. Hearing loss and heavy metal toxicity in a nicaraguan mining community: Audiological results and case reports. Audiol Neurotol. 2013;18(2):101-113. doi:10.1159/000345470.

230. Marsh D, Turner M, Smith J, Allen P, Richdale N. Fetal methylmercury study in a Peruvian fish-eating population. Neurotoxicology. 1995;16(4):717-726.

231. Astete J, Gastañaga MDC, Fiestas V, et al. Enfermedades transmisibles, salud mental y exposición a contaminantes ambientales en población aledaña al proyecto minero Las Bambas antes de la fase de explotación, Perú 2006. Rev Peru Med Exp Salud Publica. 2010;27(4):512-519.

232. Concha G, Nermell B, Vahter M. Metabolism of inorganic arsenic in children with chronic high arsenic exposure in northern Argentina. Environ Health Perspect. 1998;106(6):355-359. doi:10.1289/ehp.98106355.

233. Azcurra A, Battellino L, Calamari S, de Cattoni S, Kremer M, Lamberghini F. Estado de salud bucodental de escolares residentes en localidades abastecidas con agua de consumo humano de muy alto y muy bajo contenido de fluoruros. Rev Saude Publica. 1995;29(5):364-375.

234. Franco ÁM, Saldarriaga A, Martignon S, González M, Villa A. Flouride intake and fractional urinary fluoride excretion of Colombian preschool children. Community Dent Health. 2005;22:272-278.

235. Salas-Pereira M, Beltrán-Aguilar E, Chavarría P, Solórzano I, Horowitz H. Enamel fluorosis in 12 and 15 year-old school children in Costa Rica. Results of a national survey, 1999. Community Dent Health. 2008;25:178-184.

236. Mohan S, Tiller M, van der Voet G, Kanhai H. Mercury Exposure of Mothers and Newborns in Surinam: A Pilot Study. Clin Toxicol. 2005;43(2):101-104. doi:10.1081/CLT-200050404.

237. Ramsubhag S, Naidu R, Narinesingh D, Teelucksingh S. Urinary fluoride levels in children in a single school in Trinidad and Tobago: a preliminary investigation. West Indian Med J. 2006;55(6):440-443.

238. Kordas K, Queirolo EI, Ettinger AS, Wright RO, Stoltzfus RJ. Prevalence and predictors of exposure to multiple metals in preschool children from Montevideo, Uruguay. Sci Total Environ. 2010;408(20):4488-4494. doi:10.1002/ana.22528.Toll-like.

239. Hall D, Samms-Vaughan M, Roberson S, Onugha T, Siles R. The effects of the environment on autistic spectrum disorder in Jamaica. Rev Med Chil. 2001;50(suplemento 5).

240. Martínez-Salinas RI, Elena Leal M, Batres-Esquivel LE, et al. Exposure of children to polycyclic aromatic hydrocarbons in Mexico: assessment of multiple sources. Int Arch Occup Environ Health. 2010;83(6):617-623. doi:10.1007/s00420-009-0482-x.

241. Sánchez-Guerra M, Pelallo-Martínez N, Díaz-Barriga F, et al. Environmental polycyclic aromatic hydrocarbon (PAH) exposure and DNA damage in Mexican children. Mutat Res - Genet Toxicol Environ Mutagen. 2012;742(1-2):66-71. doi:10.1016/j.mrgentox.2011.12.006.

242. Sarnat SE, Raysoni AU, Li W-W, et al. Air pollution and acute respiratory response in a panel of asthmatic children along the U.S.-Mexico border. Environ Health Perspect. 2012;120(3):437-444. doi:10.1289/ehp.1003169.

243. Ramírez-Sánchez HU, Andrade-García MD, González-Castañeda ME, Celis-De La Rosa ADJ. Contaminantes atmosféricos y su correlación con infecciones agudas de las vías respiratorias en niños de Guadalajara, Jalisco. Salud Publica Mex. 2006;48(5):385-394. doi:10.1590/S0036-36342006000500005.

244. Hernández-Garduño E, Pérez-Neria J, Paccagnella AM, et al. Air Pollution and Respiratory Health in Mexico City. J Occup Environ Med. 1997;39(4):299-307. doi:10.1097/00043764-199704000-00006.

245. Hernández-Cadena L, Holguin F, Barraza-Villarreal A, Del Río-Navarro BE, Sienra-Monge JJ, Romieu I. Increased levels of outdoor air pollutants are associated with reduced bronchodilation in children with asthma. Chest. 2009;136(6):1529-1536. doi:10.1378/chest.08-1463.

246. Calderón-Garcidueñas L, Vincent R, Mora-Tiscareño A, et al. Elevated plasma endothelin-1 and pulmonary arterial pressure in children exposed to air pollution. Environ Health Perspect. 2007;115(8):1248-1253. doi:10.1289/ehp.9641.

247. Romieu I, Barraza-Villarreal A, Escamilla-Núñez C, et al. Dietary intake, lung function and airway inflammation in Mexico City school children exposed to air pollutants. Respir Res. 2009;10(1):122. doi:10.1186/1465-9921-10-122.

248. Sienra-Monge JJ, Ramirez-Aguilar M, Moreno-Macias H, et al. Antioxidant supplementation and nasal inflammatory responses among young asthmatics exposed to high levels of ozone. Clin Exp Immunol. 2004;138(2):317-322. doi:10.1111/j.1365-2249.2004.02606.x.

249. Gold D, Damokosh A, Pope CI, et al. Particulate and ozone pollutant effects on the respiratory function of children in southwest Mexico City. Epidemiology. 1999;10(1):8-16.

250. Romieu I, Meneses F, Sienra-Monge JJL, et al. Effects of urban air pollutants on emergency visits for childhood asthma ¡n Mexico City. Am J Epidemiol. 1995;141(6):546-553.

251. Meneses González F, Romieu I, Sienra Monge J, Huerta López J, Ruiz Velasco S. Asma en población infantil y su relación con los contaminantes ambientales aéreos de la ciudad de México. Rev Alerg Mex. 1996;43(3):66-72.

252. Calderón-Garcidueñas L, Wen-Wang L, Zhang YJ, et al. 8-hydroxy-2'-deoxyguanosine, a major mutagenic oxidative DNA lesion, and DNA strand breaks in nasal respiratory epithelium of children exposed to urban pollution. Environ Health Perspect. 1999;107(6):469-474. doi:10.1289/ehp.99107469.

253. Calderón-Garcidueñas L, Rodriguez-Alcaraz A, Garcia R, et al. Cell proliferation in nasal respiratory epithelium of people exposed to urban pollution. Carcinogenesis. 1999;20(3):383-389. doi:DOI 10.1093/carcin/20.3.383.

254. Meza Morales A, Arreguín Osuna L, Navarrete F, Huerta López J, Medina G. Características morfológicas de la mucosa nasal en niños sanos expuestos a diferentes concentraciones de contaminación atmosférica. Rev Alerg Mex. 1998;45(1):3-7.

255. Romieu I, Meneses F, Ruiz S, et al. Effects of Intermittent Ozone Exposure on Peak Expiratory Flow and Respiratory Symptoms among Asthmatic Children in Mexico City. Arch Environ Heal An Int J. 1997. doi:10.1080/00039899709602213.

256. Romieu I, Sienra-Monge JJ, Ramírez-Aguilar M, et al. Genetic polymorphism of GSTM1 and antioxidant supplementation influence lung function in relation to ozone exposure in asthmatic children in Mexico City. Thorax. 2004;59:8-10.

257. Castillejos M, Gold DRD, Damokosh AIA, et al. Acute effects of ozone on the pulmonary function of exercising schoolchildren from Mexico City. Am J Respir Crit Care Med. 1995;152(5 I):1501-1507. doi:10.1164/ajrccm.152.5.7582284.

258. Borja-Aburto VH, Loomis DP, Bangdiwala SI, Shy CM, Rascon-Pacheco RA. Ozone, Suspended Particulates, and Daily Mortality in Mexico City. Am J Epidemiol. 1997;145(3):258-268.

259. Téllez-Rojo MM, Romieu I, Polo-Peña M, Ruiz-Velasco S, Meneses-González F, Hernández-Avila M. Efecto de la contaminación ambiental sobre las consultas por infecciones respiratorias en niños de la Ciudad de México. Salud Publica Mex. 1997;39(6):513-522. doi:10.1590/S0036-36341997000600004.

260. Hernández-Cadena L, Barraza-Villarreal A, Ramírez-Aguilar M, et al. Morbilidad infantil por causas respiratorias y su relación con la contaminación atmosférica en Ciudad Juárez, Chihuahua, México. Salud Publica Mex. 2007;49(1):27-36.

261. Calderón-Garcidueñas L, Mora-Tiscareño A, Fordham LA, et al. Lung radiology and pulmonary function of children chronically exposed to air pollution. Environ Health Perspect. 2006;114(9):1432-1437. doi:10.1289/ehp.8377.

262. Calderón-Garcidueñas L, Mora-Tiscareño A, Fordham LA, et al. Respiratory damage in children exposed to urban pollution. Pediatr Pulmonol. 2003;36(2):148-161. doi:10.1002/ppul.10338.

263. Calderón-Garcidueñas L, Macías-Parra M, Hoffmann HJ, et al. Immunotoxicity and Environment: Immunodysregulation and Systemic Inflammation in Children. Toxicol Pathol. 2009;37(2):161-169. doi:10.1177/0192623308329340.

264. Escamilla-Nuñez MC, Barraza-Villarreal A, Hernandez-Cadena L, et al. Traffic-related air pollution and respiratory symptoms among asthmatic children, resident in Mexico City: the EVA cohort study. Respir Res. 2008;9(1):74-84. doi:10.1186/1465-9921-9-74.

265. Rosas I, McCartney H, Payne R, et al. Analysis of the relationships between environmental factors (aeroallergens, air pollution, and weather) and asthma emergency admissions to a hospital in Mexico City. Allergy. 1998;53(17):394-401. doi:10.1111/j.1398-9995.1998.tb03911.x.

266. Ramírez-Aguilar M, Barraza-Villarreal A, Moreno-Macías H, et al. Assessment of personal exposure to ozone in asthmatic children residing in Mexico City. Salud Publica Mex. 2008;50(1):67-75. doi:10.1590/S0036-36342008000100013.

267. Romieu I, Sienra-Monge JJ, Ramírez-Aguilar M, et al. Antioxidant supplementation and lung functions among children with asthma exposed to high levels of air pollutants. Am J Respir Crit Care Med. 2002;166(5):703-709. doi:10.1164/rccm.2112074.

268. Calderón-Garcidueñas L, Osnaya N, Rodríguez-Alcaraz A, Villarreal-Calderón A. DNA Damage in Nasal Respiratory Epithelium From Children Exposed to Urban Pollution. Environ Mol Mutagen. 1997;30:11-20.

269. Villarreal-Calderón A, Acuña H, Villareal-Calderón J, et al. Assessment of physical education time and after-school outdoor time in elementary and middle school students in south Mexico City: the dilemma between physical fitness and the adverse health effects of outdoor pollutant exposure. Arch Environ Health. 2002;57(5):450-460.

270. Calderón-Garcidueñas L, Rodriguez-Alcaraz A, Valencia-Salazar G, et al. Nasal biopsies of children exposed to air pollutants. Toxicol Pathol. 2001;29(5):558-564. doi:10.1080/019262301317226366.

271. Romieu I, Ramirez-Aguilar M, Sienra-Monge J, et al. GSTM1 and GSTP1 and respiratory health in asthmatic children exposed to ozone. Eur Respir J. 2006;28(5):953-959. doi:10.1183/09031936.06.00114905.

272. Calderón-Garcidueñas L, Mora-Tiscareño A, Ontiveros E, et al. Air pollution, cognitive deficits and brain abnormalities: a pilot study with children and dogs. Brain Cogn. 2008;68(2):117-127. doi:10.1016/j.bandc.2008.04.008.

273. Calderón-Garcidueñas L, Mora-Tiscareño A, Chung CJ, et al. Exposure to air pollution is associated with lung hyperinflation in healthy children and adolescents in Southwest Mexico City: a pilot study. Inhal Toxicol. 2000;12:537-561. doi:10.1080/089583700402905.

274. Moreno-Macías H, Dockery DW, Schwartz J, et al. Ozone exposure, vitamin C intake, and genetic susceptibility of asthmatic children in Mexico City: a cohort study. Respir Res. 2013;14:14-23. doi:10.1186/1465-9921-14-14.

275. Romieu I, Barraza-Villarreal A, Escamilla-Nuñez C, et al. Exhaled breath malondialdehyde as a marker of effect of exposure to air pollution in children with asthma. J Allergy Clin Immunol. 2008;121(4):903-909.e6. doi:10.1016/j.jaci.2007.12.004.

276. Linares B, Guizar JM, Amador N, et al. Impact of air pollution on pulmonary function and respiratory symptoms in children. Longitudinal repeated-measures study. BMC Pulm Med. 2010;10(1):62-70. doi:10.1186/1471-2466-10-62.

277. O'Neill MS, Hajat S, Zanobetti A, Ramirez-Aguilar M, Schwartz J. Impact of control for air pollution and respiratory epidemics on the estimated associations of temperature and daily mortality. Int J Biometeorol. 2005;50:121-129. doi:10.1007/s00484-005-0269-z.

278. Carbajal-Arroyo L, Miranda-Soberanis V, Medina-Ramon M, et al. Effect of PM10 and O3 on infant mortality among residents in the Mexico City Metropolitan Area: a case-crossover analysis, 1997-2005. J Epidemiol Community Health. 2010;65(8):715-721. doi:10.1136/jech.2009.101212.

279. Cañedo-Mondragó R, Eguía-Aguilar P, Pérezpeña-Díazconti M, Arenas-Huertero F. Identification of ferruginous bodies in the lungs of children and analyses of the elemental composition of fibers. Inhal Toxicol. 2013;25(9):1091-7691. doi:10.3109/08958378.2013.808288.

280. Rojas-Martinez R, Perez-Padilla R, Olaiz-Fernandez G, et al. Lung function growth in children with long-term exposure to air pollutants in Mexico City. Am J Respir Crit Care Med. 2007;176(4):377-384. doi:10.1164/rccm.200510-1678OC.

281. Barraza-Villarreal A, Sunyer J, Hernández-Cadena L, et al. Air pollution, airway inflammation, and lung function in a cohort study of Mexico City schoolchildren. Environ Health Perspect. 2008;116(6):832-838. doi:10.1289/ehp.10926.

282. Barraza-Villarreal A, Escamilla-Nuñez MC, Hernández-Cadena L, et al. Elemental carbon exposure and lung function in schoolchildren from Mexico City. Eur Respir J. 2011;38(3):548-552. doi:10.1183/09031936.00111410.

283. Romieu I, Ramirez-Aguilar M, Moreno-Macias H, et al. Infant mortality and air pollution: modifying effect by social class. J Occup Environ Med. 2004;46(12):1210-1216. doi:10.1097/01.jom.0000147224.35351.08.

284. Romieu I, Meneses F, Ruiz S, et al. Effects of air pollution on the respiratory health of asthmatic children living in Mexico City. Am J Respir Crit Care Med. 1996;154(2 Pt 1):300-307. doi:10.1164/ajrccm.154.2.8756798.

285. Calderón-Garcidueñas L, Villarreal-Calderon R, Valencia-Salazar G, et al. Systemic inflammation, endothelial dysfunction, and activation in clinically healthy children exposed to air pollutants. Inhal Toxicol. 2008;20(5):499-506. doi:10.1080/08958370701864797.

286. Hernández-Cadena L, Téllez-Rojo MM, Sanín-Aguirre LH, Lacasaña-Navarro M, Campos A, Romieu I. Relacion entre consultas a urgencias por enfermedad respiratoria y contaminacion atmosferica en Ciudad Juarez, Chihuahua. Salud Publica Mex. 2000;42(4):288-297. doi:10.1590/S0036-36342000000400003.

287. Holguin F, Flores S, Ross Z, et al. Traffic-related exposures, airway function, inflammation, and respiratory symptoms in children. Am J Respir Crit Care Med. 2007;176(12):1236-1242. doi:10.1164/rccm.200611-1616OC.

288. Santos-Burgoa C, Rojas-Bracho L, Ramírez-Sánchez A, et al. Modelaje de exposición a partículas en población general y riesgo de enfermedad respiratoria. Gac Med Mex. 134(4):407-418.

289. Loomis D, Castillejos M, Gold DR, McDonnell W, Borja-Aburto VH. Air pollution and infant mortality in Mexico City. Epidemiology. 1999;10:118-123.

290. Bell ML, O'Neill MS, Ranjit N, Borja-Aburto VH, Cifuentes LA, Gouveia NC. Vulnerability to heat-related mortality in Latin America: a case-crossover study in São Paulo, Brazil, Santiago, Chile and Mexico City, Mexico. Int J Epidemiol. 2008;37(4):796-804. doi:10.1093/ije/dyn094.

291. Gouveia N, Bremner S, Novaes H. Association between ambient air pollution and birth weight in São Paulo, Brazil. J Epidemiol Community Health. 2004;58(2):11-17. doi:0143-005X.

292. Conceicao GM, Miraglia SG, Kishi HS, Saldiva PH, Singer JM. Air pollution and child mortality: a time-series study in Sao Paulo, Brazil. Environ Health Perspect. 2001;109(sup 3):347-350.

293. Correira-Deur JE de M, Claudio L, Takimoto Imazawa A, et al. Variations in Peak Expiratory Flow Measurements Associated To Air Pollution and Allergic Sensitization in Children in Sao Paulo, Brazil. Am J Ind Med. 2012;55(12):1087-1098. doi:10.1002/ajim.22060.VARIATIONS.

294. Gouveia N, Fletcher T. Respiratory diseases in children and outdoor air pollution in São Paulo, Brazil: a time series analysis. Occup Environ Med. 2000;57:477-483.

295. Nascimento LFC, Moreira DA. Are environmental pollutants risk factors for low birth weight? Cad Saude Publica. 2009;25(8):1791-1796. http://www.scielosp.org/scielo.php?script=sci_arttext&pid=S0102-311X2009000800015.

296. Albuquerque De Castro H, Faria da Cunha M, Mendonça GA e S, et al. Effect of air pollution on lung function in schoolchildren in Rio de Janeiro, Brazil. Rev Saude Publica. 2009;43(1):26-34. doi:10.1590/S0034-89102009000100004.

297. Farhat SCL, Almeida MB, Silva-Filho LVRF, Farhat J, Rodrigues JC, Braga ALFAL. Ozone is associated with an increased risk of respiratory exacerbations in patients with cystic fibrosis. Chest. 2013;144(4):1186-1192. doi:10.1378/chest.12-2414.

298. Vieira SE, Stein RT, Ferraro AA, et al. Urban air pollutants are significant risk factors for asthma and pneumonia in children: the influence of location on the measurement of pollutants. Arch Bronconeumol. 2012;48(11):389-395. doi:10.1016/j.arbres.2012.05.005.

299. Romao R, Amador Pereira LA, Nascimento Saldiva PH, Matias Pinheiro P, Ferreira Braga AL, Martins LC. The relationship between low birth weight and exposure to inhalable particulate matter. Cad Saude Publica. 2013;29(6):1101-1108.

300. Braga ALFL, Saldiva PHHN, Pereira LAAA, et al. Health Effects of Air Pollution Exposure on Children and Adolescents in Sao Paulo, Brazil. Pediatr Pulmonol. 2001;31:106-113. doi:10.1002/1099-0496(200102)31.

301. Luiz J, Rios M, Boechat JL, et al. Atmospheric pollution and the prevalence of asthma: study among schoolchildren of 2 areas in Rio de Janeiro, Brazil. Ann Allergy, Asthma Immunol. 2004;92(6):629-634. doi:10.1016/S1081-1206(10)61428-7.

302. Ribeiro H, Alves Cardoso MR, Cardoso MRA. Air pollution and children's health in São Paulo (1986-1998). Soc Sci Med. 2003;57(11):2013-2022. doi:10.1016/S0277-9536(03)00068-6.

303. Gouveia N, Fletcher T. Time series analysis of air pollution and mortality: effects by cause, age and socioeconomic status. J Epidemiol Community Heal. 2000;54(10):750-755. doi:10.1136/jech.54.10.750.

304. De Sousa Zanotti Stagliorio Coelho M, Teixeiṛa Goncalves FL, Dias De Oliveira Latorre MDR. Statistical analysis aiming at predicting respiratory tract disease hospital admissions from environmental variables in the city of São Paulo. J Environ Public Health. 2010;2010:1-11. doi:10.1155/2010/209270.

305. Lopes de Moraes AC, Ignotti E, Netto PA, Viana Jacobson LDS, Castro H, de Souza Hacon S. Wheezing in children and adolescents living next to a petrochemical plant in Rio Grande do Norte, Brazil. J Pediatr (Rio J). 2010;86(4):337-344. doi:10.2223/JPED.2020.

306. Cançado JEDE, Saldiva PHHN, Pereira LAAA, et al. The impact of sugar cane-burning emissions on the respiratory system of children and the elderly. Environ Health Perspect. 2006;114(5):725-729. doi:10.1289/ehp.8485.

307. Sousa S, Pires J, Martins E, Fortes J, Alvim-Ferraz M, Martins F. Short-term effects of air pollution on respiratory morbidity at Rio de Janeiro — Part II: health assessment. Environ Int. 2012;43:1-5. doi:10.1016/j.envint.2012.02.004.

308. Albuquerque de Castro H, Hacon S, Argento R, et al. Air pollution and respiratory diseases in the Municipality of Vitória, Espírito Santo State, Brazil. Cad Saude Publica. 2007;23(sup 4):S630-S642. doi:10.1590/S0102-311X2007001600023.

309. Moura M, Leite Junger W, Azevedo e Silva Mendonca G, Ponce de Leon A. Air quality and emergency pediatric care for symptoms of bronchial obstruction categorized by age bracket in Rio de Janeiro, Brazil. Cad Saude Publica. 2009;25(3):635-644.

310. Amancio CT, Nascimento LFC, Amancio TT. Environmental pollutants and odds of hospitalization for asthma in children - São José Dos Campos, Brazil, in the years 2004-2005. J Hum Growth Dev. 2012.

311. Lin C, Martins M, Farhat S, et al. Air pollution and respiratory illness of children in São Paulo, Brazil. Pediatr Perinat Epidemiol. 1999;13(4):475-488. doi:10.1046/j.1365-3016.1999.00210.x.

312. Marcilio I, Gouveia N. Quantifyng the impact of air pollution on the urban population in Brazil. Cad Saude Publica. 2007;23(sup 4):S529-S536.

313. Trolez Amâncio C, Costa Nascimento LF. Asthma and ambient pollutants: a time series study. Rev Assoc Med Bras. 2012;58(3):302-307. doi:10.1590/S0104-42302012000300009.

314. Peneluppi de Medeiros AP, Gouveia N, Pérez Machado RP, et al. Traffic-Related Air Pollution and Perinatal Mortality: a Case–Control Study. Environ Health Perspect. 2009;117(1):127-132. doi:10.1289/ehp.11679.

315. Lin C, Pereira L, Nishioka D, Conceição G, Braga A, Saldiva P. Air pollution and neonatal deaths in Sao Paulo, Brazil. Brazilian J Med Biol Res. 2004;37:765-770.

316. Northcross A, Chowdhury Z, McCracken J, Canuz E, Smith KR. Estimating personal PM2.5 exposures using CO measurements in Guatemalan households cooking with wood fuel. J Environ Monit. 2010;12(4):873-878. doi:10.1039/b916068j.

317. McCracken JP, Schwartz J, Bruce N, Mittleman M, Ryan LM, Smith KR. Combining Individual- and Group-Level Exposure Information: child carbon monoxide in the Guatemala woodstove randomized control trial. Epidemiology. 2009;20(1):127-136. doi:10.1097/EDE.0b013e31818ef327.

318. Smith KR, McCracken JP, Weber MW, et al. Effect of reduction in household air pollution on childhood pneumonia in Guatemala (RESPIRE): a randomised controlled trial. Lancet. 2011;378(9804):1717-1726. doi:10.1016/S0140-6736(11)60921-5.

319. Dix-Cooper L, Eskenazi B, Romero C, Balmes J, Smith KR. Neurodevelopmental performance among school age children in rural Guatemala is associated with prenatal and postnatal exposure to carbon monoxide, a marker for exposure to woodsmoke. Neurotoxicology. 2012;33(2):246-254. doi:10.1016/j.neuro.2011.09.004.

320. Thompson LM, Bruce N, Eskenazi B, Diaz A, Pope D, Smith KR. Impact of reduced maternal exposures to wood smoke from an introduced chimney stove on newborn birth weight in rural Guatemala. Environ Health Perspect. 2011. doi:10.1289/ehp.1002928.

321. Smith KR, McCracken JP, Thompson L, et al. Personal child and mother carbon monoxide exposures and kitchen levels: methods and results from a randomized trial of woodfired chimney cookstoves in Guatemala (RESPIRE). J Expo Sci Environ Epidemiol. 2010;20(5):406-416. doi:10.1038/jes.2009.30.

322. Schei MA, Hessen JO, Smith KR, Bruce N, Mccracken J, Lopez V. Childhood asthma and indoor woodsmoke from cooking in Guatemala. J Expo Anal Environ Epidemiol. 2004;14(Suppl. 1):S110-S117. doi:10.1038/sj.jea.7500365.

323. Boy E, Bruce N, Delgado H. Birth weight and exposure to kitchen wood smoke during pregnancy in rural Guatemala. Environ Health Perspect. 2002;110(1):109-114. doi:10.1289/ehp.02110109.

324. Muñoz F, Carvalho MS. Efecto del tiempo de exposición a PM10 en las urgencias por bronquitis aguda. Cad Saude Publica. 2009;25(3):529-539.

325. Ilabaca M, Olaeta I, Campos E, Villaire J, Tellez-Rojo MM, Romieu I. Association between levels of fine particulate and emergency visits for pneumonia and other respiratory illnesses among children in Santiago, Chile. J Air Waste Manag Assoc. 1999;49(9):154-163. doi:10.1080/10473289.1999.10463879.

326. Sánchez J, Romieu I, Ruiz S, Pino P, Gutiérrez M. Efectos agudos de las partículas respirables y del dióxido de azufre sobre la salud respiratoria en niños del área industrial de Puchuncaví, Chile. Rev Panam Salud Publica. 1999;6(6):384-391.

327. Pino P, Walter T, Oyarzun M, Villegas R, Romieu I. Fine Particulate Matter and Wheezing Illnesses in the First Year of Life. Epidemiology. 2004;15(6):702-708. doi:10.1097/01.ede.0000142153.28496.d0.

328. González R N, Torres-Avilés F, Carrasco P E, Salas P F, Pérez B F. Estudio temporal de diabetes mellitus tipo 1 en Chile: asociación con factores ambientales durante el período 2000-2007. Rev Med Chil. 2013;141:595-601.

329. Ostro BD, Eskeland GS, Sanchez JM, Feyzioglu T. Air pollution and health effects: a study of medical visits among children in Santiago, Chile. Environ Health Perspect. 1999;107:69-73.

330. Pino P, Oyarzún M, Walter T, von Baer D, Romieu I. Contaminación aérea intradomiciliaria en el área sur-oriente de Santiago. Rev Med Chil. 1998;126:367-374.

331. Castro-Jiménez MÁ, Orozco-Vargas LC. Parental exposure to carcinogens and risk for childhood acute lymphoblastic leukemia, Colombia, 2000-2005. Prev Chronic Dis. 2011;8(5):A106. http://www.pubmedcentral.nih.gov/articlerender.fcgi?artid=3181179&tool=pmcentrez&rendertype=abstract.

332. Rodriguez-Villamizar LA, Castro-Ortiz H, Rey-Serrano JJ. The effects of air pollution on respiratory health in susceptible populations: a multilevel study in Bucaramanga, Colombia. Cad Saude Publica. 2012;28(4):749-757. doi:10.1590/S0102-311X2012000400014.

333. Rodríguez Villamizar LA, Herrera López AB, Castro Ortiz H, Niederbacher Velázquez J, Vera Cala LM. Incidence of respiratory symptoms and the association with air pollution in preschoolers: a multilevel analysis. Cad Saude Publica. 2010;26(7):1411-1418.

334. Rodríguez L, Rey J, Herrera A, et al. Prevalencia de síntomas respiratorios indicativos de asma y asociación con contaminación atmosférica en preescolares de Bucaramanga, Colombia. Biomédica. 2010;30:15-22. doi:10.7705/biomedica.v30i1.148.

335. Estrella B, Estrella R, Oviedo J, et al. Acute respiratory diseases and carboxyhemoglobin status in school children of Quito, Ecuador. Environ Health Perspect. 2005;113(5):607-611. doi:10.1289/ehp.7494.

336. Rinne ST, Rodas EJ, Bender BS, et al. Relationship of pulmonary function among women and children to indoor air pollution from biomass use in rural Ecuador. Respir Med. 2006;100(7):1208-1215. doi:10.1016/j.rmed.2005.10.020.Relationship.

337. Rinne ST, Rodas EJ, Rinne ML, Simpson JM, Glickman LT. Use of biomass fuel is associated with infant mortality and child health in trend analysis. Am J Trop Med Hyg. 2007;76(3):585-591.

338. Harris AM, Sempértegui F, Estrella B, et al. Air pollution and anemia as risk factors for pneumonia in Ecuadorian children: a retrospective cohort analysis. Environ Heal. 2011;10(1):93-100. doi:10.1186/1476-069X-10-93.

339. Yucra S, Tapia V, Steenland K, Naeher LP, Gonzales GFG. Maternal exposure to biomass smoke and carbon monoxide in relation to adverse pregnancy outcome in two high altitude cities of Peru. Environ Res. 2014;130:29-33. doi:10.1016/j.envres.2014.01.008.

340. Castañeda JL, Kheirandish-Gozal L, Gozal D, Accinelli RA, The Pampa Cangallo Instituto de Investigaciones de la Altura Research. Effect of reductions in biomass fuel exposure on symptoms of sleep apnea in children living in the peruvian andes: a preliminary field study. Pediatr Pulmonol. 2013;48(10):996-999. doi:10.1002/ppul.22720.

341. Robinson CL, Baumann LM, Romero K, et al. Effect of urbanisation on asthma, allergy and airways inflammation in a developing country setting. Thorax. 2011;66(12):1051-1057. doi:10.1136/thx.2011.158956.

342. Bautista LE, Correa A, Baumgartner J, Breysse P, Matanoski GM. Indoor charcoal smoke and acute respiratory infections in young children in the Dominican Republic. Am J Epidemiol. 2009;169(5):572-580. doi:10.1093/aje/kwn372.

343. Hubbell AM, Jareczek FJ, Vonnahme L, Hockenberry JM, Buresh C. Smoke exposure among women in Haiti: the case for improved stoves. Glob Public Health. 2013;8(7):822-830. doi:10.1080/17441692.2013.815793.

344. Wichmann FA, Müller A, Busi LE, et al. Increased asthma and respiratory symptoms in children exposed to petrochemical pollution. J Allergy Clin Immunol. 2009;123(3):632-638. doi:10.1016/j.jaci.2008.09.052.

345. Rivas Riveros E, Barrios C S, Dorner P A, Osorio S X. Fuentes de contaminación intradomiciliaria y enfermedad respiratoria en jardines infantiles y salas cunas de Temuco y Padre Las Casas, Chile. Rev Med Chil. 2008;136(6):767-774. doi:10.4067/S0034-98872008000600013.

346. Torres-Arreola L, Berkowitz G, Torres-Sánchez L, et al. Preterm birth in relation to maternal organochlorine serum levels. Ann Epidemiol. 2003;13(3):158-162. doi:10.1016/S1047-2797(02)00424-6.

347. Meza-Montenegro MM, Valenzuela-Quintanar AI, Balderas-Cortés JJ, et al. Exposure assessment of organochlorine pesticides, arsenic, and lead in children from the major agricultural areas in Sonora, Mexico. Arch Environ Contam Toxicol. 2013. doi:10.1007/s00244-012-9846-4.

348. Cupul-Uicab LA, Hernández-Ávila M, Terrazas-Medina EA, Pennell ML, Longnecker MP. Prenatal exposure to the major DDT metabolite 1,1-dichloro-2,2-bis(p-chlorophenyl)ethylene (DDE) and growth in boys from Mexico. Environ Res. 2010. doi:10.1016/j.envres.2010.06.001.

349. Yáñez L, Ortiz-Pérez D, Batres LE, Borja-Aburto VH, Díaz-Barriga F. Levels of Dichlorodiphenyltrichloroethane and Deltamethrin in Humans and Environmental Samples in Malarious Areas of Mexico. Environ Res. 2002. doi:10.1006/enrs.2002.4333.

350. Pérez-Maldonado IN, Trejo-Acevedo A, Pruneda-Alvarez LG, Gaspar-Ramirez O, Ruvalcaba-Aranda S, Perez-Vazquez FJ. DDT, DDE, and 1-hydroxypyrene levels in children (in blood and urine samples) from Chiapas and Oaxaca, Mexico. Environ Monit Assess. 2013;185(11):9287-9293. doi:10.1007/s10661-013-3251-y.

351. Herrero-Mercado M, Waliszewski SMS, Caba M, et al. Organochlorine pesticide levels in umbilical cord blood of newborn in Veracruz, Mexico. Bull Environ Contam Toxicol. 2010;85(4):367-371. doi:10.1007/s00128-010-0108-8.

352. Trejo-Acevedo A, Rivero-Pérez N, Flores-Ramírez R, Díaz-Barriga F, Ochoa Angeles C, Pérez-Maldonado I. Assessment of persistent organic pollutants levels in blood samples from Quintana Roo, Mexico. Int J Hyg Environ Health. 2013;216(3):284-289. doi:10.1016/j.ijheh.2012.09.004.

353. Torres-Arreola L, López-Carrillo L, Torres-Sánchez L, et al. Levels of dichloro-dyphenyl-trichloroethane (DDT) metabolites in maternal milk and their determinant factors. Arch Environ Health. 1999;54(2):124-129. doi:10.1086/250095.

354. Torres-Sánchez L, Rothenberg SJ, Schnaas L, et al. In Utero p,p'-DDE exposure and infant neurodevelopment: a perinatal cohort in Mexico. Environ Health Perspect. 2007;115(3):435-439. doi:10.1289/ehp9566.

355. Waliszewski SMS, Aguirre AA, Infanzon RMR, Silva CS, Siliceo J. Organochlorine pesticide levels in maternal adipose tissue, maternal blood serum, umbilical blood serum, and milk from inhabitants of Veracruz, Mexico. Arch Environ Contam Toxicol. 2001;40(3):432-438. doi:10.1007/s002440010194.

356. Koepke R, Warner M, Petreas M, et al. Serum DDT and DDE levels in pregnant women of Chiapas, Mexico. Arch Environ Health. 2004;59(11):559-565.

357. Martínez-Salinas RI, Pérez-Maldonado IN, Batres-Esquivel LE, Flores-Ramírez R, Díaz-Barriga F. Assessment of DDT, DDE, and 1-hydroxypyrene levels in blood and urine samples in children from Chiapas Mexico. Environ Sci Pollut Res. 2012;19(7):2658-2666. doi:10.1007/s11356-012-0758-7.

358. Pérez-Maldonado IN, Athanasiadou M, Yáñez L, González-Amaro R, Bergman A, Díaz-Barriga F. DDE-induced apoptosis in children exposed to the DDT metabolite. Sci Total Environ. 2006;370(2-3):343-351. doi:10.1016/j.scitotenv.2006.06.026.

359. Bustamante Montes LP, Waliszewski S, Hernández-Valero M, Sanín-Aguirre L, Infanzón-Ruiz RM, García Jañas A. Exposición prenatal a los plaguicidas organoclorados y criptorquidia. Cienc e Saude Coletiva. 2010;15(supl. 1):1169-1174. doi:10.1590/S1413-81232010000700025.

360. Herrera-Portugal C, Ochoa H, Franco-Sánchez G, Yáñez L, Díaz-Barriga F. Environmental pathways of exposure to DDT for children living in a malarious area of Chiapas, Mexico. Environ Res. 2005;99(2):158-163. doi:10.1016/j.envres.2005.03.010.

361. Waliszewski SM, Aguirre AA, Infanzón RM, Siliceo J. Carry-over of persistent organochlorine pesticides through placenta to fetus. Salud Publica Mex. 2000;42(5):384-390. doi:10.1590/S0036-36342000000500003.

362. Domínguez-Cortinas G, Díaz-Barriga F, Martínez-Salinas RI, Cossío P, Pérez-Maldonado IN. Exposure to chemical mixtures in Mexican children: high-risk scenarios. Environ Sci Pollut Res. 2013;20(1):351-357. doi:10.1007/s11356-012-0933-x.

363. Trejo-Acevedo A, Rivero-Pérez NE, Flores-Ramírez R, Orta-García ST, Varela-Silva JA, Pérez-Maldonado IN. Assessment of the levels of persistent organic pollutants and 1-hydroxypyrene in blood and urine samples from Mexican children living in an endemic malaria area in Mexico. Bull Environ Contam Toxicol. 2012. doi:10.1007/s00128-012-0593-z.

364. Pardío VTV, Waliszewski SMS, Aguirre AA, et al. DDT and its metabolites in human milk collected in Veracruz City and suburban areas (Mexico). Bull Environ Contam Toxicol. 1998;60(6):852-857. doi:10.1007/s001289900705.

365. Elvia LF, Sioban HD, Hernández PB, Sánchez Carrillo C. Organochlorine pesticide exposure in rural and urban areas in Mexico. J Expo Anal Environ Epidemiol. 2000;10:394-399.

366. Díaz-Barriga Martínez F, Trejo-Acevedo A, Betanzos AF, et al. Assessment of DDT and DDE levels in soil, dust, and blood samples from Chihuahua, Mexico. Arch Environ Contam Toxicol. 2012;62(2):351-358. doi:10.1007/s00244-011-9700-0.

367. Torres-Sánchez L, Schnaas L, Rothenberg SJ, et al. Prenatal p,p’-DDE exposure and neurodevelopment among children 3.5-5 years of age. Environ Health Perspect. 2013;121(2):263-268. doi:10.1289/ehp.1205034.

368. Torres-Sanchez L, Zepeda M, Cebrián ME, et al. Dichlorodiphenyldichloroethylene exposure during the first trimester of pregnancy alters the anal position in male infants. In: Annals of the New York Academy of Sciences.; 2008. doi:10.1196/annals.1454.004.

369. Longnecker MP, Gladen BC, Cupul-Uicab LA, et al. In utero exposure to the antiandrogen 1,1-dichloro-2,2-bis(p-chlorophenyl) ethylene (DDE) in relation to anogenital distance in male newborns from Chiapas, México. Am J Epidemiol. 2007;165(9):1015-1022. doi:10.1093/aje/kwk109.

370. Torres-Sánchez L, Schnaas L, Cebrián ME, et al. Prenatal dichlorodiphenyldichloroethylene (DDE) exposure and neurodevelopment: A follow-up from 12 to 30 months of age. Neurotoxicology. 2009;30(6):1162-1165. doi:10.1016/j.neuro.2009.08.010.

371. Rodríguez-Dozal S, Riojas Rodríguez H, Hernández-Ávila M, et al. Persistent organic pollutant concentrations in first birth mothers across Mexico. J Expo Sci Environ Epidemiol. 2012;22(1):60-69. doi:10.1038/jes.2011.31.

372. Trejo-Acevedo A, Díaz-Barriga F, Carrizales L, et al. Exposure assessment of persistent organic pollutants and metals in Mexican children. Chemosphere. 2009;74:974-980. doi:10.1016/j.chemosphere.2008.10.030.

373. Flores-Luévano S, Farías P, Hernández M, et al. DDT DDE concentrations and risk of hypospadias. Un estudio piloto de casos y controles. Salud Publica Mex. 2003;45:431-438.

374. Garced S, Torres-Sánchez L, Cebrián ME, Claudio L, López-Carrillo L. Prenatal dichlorodiphenyldichloroethylene (DDE) exposure and child growth during the first year of life. Environ Res. 2012. doi:10.1016/j.envres.2011.12.002.

375. Bahena-Medina LA, Torres-Sánchez L, Schnaas L, et al. Neonatal neurodevelopment and prenatal exposure to dichlorodiphenyldichloroethylene (DDE): A cohort study in Mexico. J Expo Sci Environ Epidemiol. 2011. doi:10.1038/jes.2011.25.

376. Cupul-Uicab LA, Gladen BC, Hernández-Ávila M, Weber JP, Longnecker MP. DDE, a degradation product of DDT, and duration of lactation in a highly exposed area of Mexico. Environ Health Perspect. 2008;116(2):179-183. doi:10.1289/ehp.10550.

377. Gaspari L, Sampaio DRD, Paris F, et al. High prevalence of micropenis in 2710 male newborns from an intensive-use pesticide area of Northeastern Brazil. Int J Androl. 2012;35(3):253-264. doi:10.1111/j.1365-2605.2011.01241.x.

378. Freire C, Koifman RJ, Sarcinelli P, Rosa AC, Clapauch R, Koifman S. Long term exposure to organochlorine pesticides and thyroid function in children from Cidade dos Meninos, Rio de Janeiro, Brazil. Environ Res. 2012. doi:10.1016/j.envres.2012.06.009.

379. Azeredo A, Torres JPMP, de Freitas Fonseca M, et al. DDT and its metabolites in breast milk from the Madeira River basin in the Amazon, Brazil. Chemosphere. 2008;73(1 supplement):S246-S251. doi:10.1016/j.chemosphere.2007.04.090.

380. Rudge CVC, Sandanger T, Röllin HB, et al. Levels of selected persistent organic pollutants in blood from delivering women in seven selected areas of São Paulo State, Brazil. Environ Int. 2012. doi:10.1016/j.envint.2011.07.006.

381. Dorea JJG, Cruz-Granja AAC, Lacayo-Romero MML, Cuadra-Leal J. Perinatal Metabolism of Dichlorodiphenyldichloroethylene in Nicaraguan Mothers. Environ Res. 2001;86(3):229-237. doi:10.1006/enrs.2001.4277.

382. Lacayo Romero ML, Cruz Granja AC, Dorea JG. Concentrations of organochlorine pesticides in milk of nicaraguan mothers. Arch Environ Health. 2000;55(4):274-278. doi:10.1080/00039890009603418.

383. Cuadra SN, Linderholm L, Athanasiadou M, Jakobsson K. Persistent Organochlorine Pollutants in Children Working at a Waste-disposal Site and in Young Females with High Fish

Consumption in Managua, Nicaragua. AMBIO A J Hum Environ. 2006;35(3):109-116. doi:10.1579/0044-7447(2006)35[109:POPICW]2.0.CO;2.

384. Crump C, Michaud P, Téllez R, et al. Does perchlorate in drinking water affect thyroid function in new borns or school-age children? J Occup Environ Med. 2000;42(6):603-612.

385. Rojas-Squella X, Santos L, Baumann W, et al. Presence of organochlorine pesticides in breast milk samples from Colombian women. Chemosphere. 2013;91(6):733-739. doi:10.1016/j.chemosphere.2013.02.026.

386. Pérez-Maldonado IN, Trejo A, Ruepert C, et al. Assessment of DDT levels in selected environmental media and biological samples from Mexico and Central America. Chemosphere. 2010;78(10):1244-1249. doi:10.1016/j.chemosphere.2009.12.040.

387. Hernández Contreras N, Chang Camero Y, Santana Suárez Y, Machado Martínez E, Martinez Izquierdo AM, Pui Vazquez L de la C. Uso deliberado de diversos productos para el control de Pediculus capitis (De Geer, 1778), por padres o tutores de niños de escuelas primarias. Rev Cubana Med Trop. 2010;62(2):119-124.

388. Balluz L, Moll D, Martinez MGD, et al. Environmental pesticide exposure in Honduras following hurricane Mitch. Bull World Health Organ. 2001;79(4):288-295. doi:10.1590/S0042-96862001000400004.

389. Levario-Carrillo M, Amato D, Ostrosky-Wegman P, González-Horta C, Corona Y, Sanin LH. Relation between pesticide exposure and intrauterine growth retardation. Chemosphere. 2004;55(10):1421-1427. doi:10.1016/j.chemosphere.2003.11.027.

390. Ortiz-Pérez MD, Torres-Dosal A, Batres LE, et al. Environmental health assessment of deltamethrin in a malarious area of Mexico: Environmental persistence, toxicokinetics, and genotoxicity in exposed children. Environ Health Perspect. 2005;113(6):782-786. doi:10.1289/ehp.7652.

391. Gamlin J, Romo PD, Hesketh T, Diaz Romo P, Hesketh T. Exposure of young children working on Mexican tobacco plantations to organophosphorous and carbamic pesticides, indicated by cholinesterase depression. Child Care Health Dev. 2007;33(3):246-248. doi:10.1111/j.1365-2214.2006.00702.x.

392. Medina-Carrillo L, Rivas-Solis F, Fernández-Argüelles R. Risk for congenital malformations in pregnant women exposed to pesticides in the state of Nayarit, Mexico. Ginecol Obstet Mex. 2002;70:538-544.

393. Moreno-Banda G, Blanco-Muñoz J, Lacasaña M, et al. Maternal exposure to floricultural work during pregnancy, PON1 Q192R polymorphisms and the risk of low birth weight. Sci Total Environ. 2009;407(21):5478-5485. doi:10.1016/j.scitotenv.2009.06.033.

394. Handal AJ, Harlow SD, Breilh J, Lozoff B. Occupational Exposure to Pesticides During Pregnancy and Neurobehavioral Development of Infants and Toddlers. Epidemiology. 2008;19:851-859. doi:10.1097/EDE.0b013e318187cc5d.

395. Harari R, Julvez J, Murata K, et al. Neurobehavioral deficits and increased blood pressure in school-age children prenatally exposed to pesticides. Environ Health Perspect. 2010;118(6):890-896. doi:10.1289/ehp.0901582.

396. Suarez-Lopez JRJ, Jacobs DJDR, Himes JJH, Alexander BBH, Lazovich D, Gunnar M. Lower acetylcholinesterase activity among children living with flower plantation workers. Environ Res. 2012;114:53-59. doi:10.1016/j.envres.2012.01.007.Lower.

397. Handal AJ, Lozoff B, Breilh J, Harlow SD. Effect of community of residence on neurobehavioral development in infants and young children in a flower-growing region of Ecuador. Environ Health Perspect. 2007. doi:10.1289/ehp.9261.

398. Handal AJ, Lozoff B, Breilh J, Harlow SD. Neurobehavioral Development in Children With Potential Exposure to Pesticides. Epidemiology. 2007;18(3):312-320. doi:10.1097/01.ede.0000259983.55716.bb.

399. Bulgaroni V, Rovedatti MG, Sabino G, Magnarelli G. Organophosphate pesticide environmental exposure: analysis of salivary cholinesterase and carboxilesterase activities in preschool children and their mothers. Environ Monit Assess. 2012;184(5):3307-3314. doi:10.1007/s10661-011-2190-8.

400. Monge P, Wesseling C, Guardado J, et al. Parental occupational exposure to pesticides and the risk of childhood leukemia in Costa Rica. Scand J Work Environ Health. 2007;33(4):293-303. doi:10.5271/sjweh.1146.

401. Azaroff L, Neas L. Acute health effects associated with nonoccupational pesticide exposure in rural El Salvador. Environ Res. 1999;80(2):158-164. doi:10.1006/enrs.1998.3878.

402. Bustamante Montes LP, García Fábila MM, Martínez Romero E, et al. Exposición a ftalatos por procedimientos médicos en varones recién nacidos. Rev Int Contam Ambient. 2005;21(2):63-69.

403. Bustamante-Montes LP, Lizama-Soberanis B, Vázquez-Moreno F, et al. Exposición infantil a plastificantes potencialmente tóxicos en productos de uso oral. Salud Publica Mex. 2004;46(6):501-508. doi:10.1590/S0036-36342004000600004.

404. Pérez-Maldonado IN, Ramírez-Jiménez M del R, Martínez-Arévalo LP, et al. Exposure assessment of polybrominated diphenyl ethers (PBDEs) in Mexican children. Chemosphere. 2009;75(9):1215-1220. doi:10.1016/j.chemosphere.2009.01.083.

405. Téllez-Rojo MM, Cantoral A, Cantonwine DE, et al. Prenatal urinary phthalate metabolites levels and neurodevelopment in children at two and three years of age. Sci Total Environ. 2013;461-462:386-390. doi:10.1016/j.scitotenv.2013.05.021.

406. Paumgartten F, Cruz C, Chahoud I, Palavinskas R, Mathar W. PCDDs, PCDFs, PCBs, and other organochlorine compounds in human milk from Rio de Janeiro, Brazil. Environ Res. 2000;83(3):293-297. doi:10.1006/enrs.2000.4062.

407. Téllez Téllez R, Michaud Chacón P, Reyes Abarca C, et al. Long-Term Environmental Exposure to Perchlorate Through Drinking Water and Thyroid Function During Pregnancy and the Neonatal Period. Thyroid. 2005;15(9):963-975. doi:10.1089/thy.2005.15.963.

408. San Sebastián M, Armstrong B, Stephens C. Outcomes of pregnancy among women living in the proximity of oil fields in the Amazon basin of Ecuador. Int J Occup Environ Health. 2002;8(4):312-319. doi:10.1179/oeh.2002.8.4.312.

409. Athanasiadou M, Cuadra SN, Marsh G, Bergman Å, Jakobsson K. Polybrominated diphenyl ethers (PBDEs) and bioaccumulative hydroxylated PBDE metabolites in young humans from Managua, Nicaragua. Environ Health Perspect. 2008. doi:10.1289/ehp.10713.
